# A comparative genomics perspective on the genetic content of the alkaliphilic haloarchaeon *Natrialba magadii* ATCC 43099^T^

**DOI:** 10.1186/1471-2164-13-165

**Published:** 2012-05-04

**Authors:** Shivakumara Siddaramappa, Jean F Challacombe, Rosana E DeCastro, Friedhelm Pfeiffer, Diego E Sastre, María I Giménez, Roberto A Paggi, John C Detter, Karen W Davenport, Lynne A Goodwin, Nikos Kyrpides, Roxanne Tapia, Samuel Pitluck, Susan Lucas, Tanja Woyke, Julie A Maupin-Furlow

**Affiliations:** 1 DOE Joint Genome Institute, Los Alamos National Laboratory, Los Alamos, NM, 87545, USA; 2 Instituto de Investigaciones Biológicas, Facultad de Ciencias Exactas y Naturales, Universidad Nacional de Mar del Plata, Funes 3250 4to Nivel, Mar del Plata, 7600, Argentina; 3 Department of Membrane Biochemistry, Max Planck Institute of Biochemistry, Am Klopferspitz 18, D-82152, Martinsried, Germany; 4 DOE Joint Genome Institute, Walnut Creek, CA, 94598, USA; 5 Department of Microbiology and Cell Science, University of Florida, Bldg. 981, Museum Rd., P.O. Box 110700, Gainesville, FL, 32611-0700, USA

## Abstract

**Background:**

*Natrialba magadii* is an aerobic chemoorganotrophic member of the *Euryarchaeota* and is a dual extremophile requiring alkaline conditions and hypersalinity for optimal growth. The genome sequence of *Nab. magadii* type strain ATCC 43099 was deciphered to obtain a comprehensive insight into the genetic content of this haloarchaeon and to understand the basis of some of the cellular functions necessary for its survival.

**Results:**

The genome of *Nab. magadii* consists of four replicons with a total sequence of 4,443,643 bp and encodes 4,212 putative proteins, some of which contain peptide repeats of various lengths. Comparative genome analyses facilitated the identification of genes encoding putative proteins involved in adaptation to hypersalinity, stress response, glycosylation, and polysaccharide biosynthesis. A proton-driven ATP synthase and a variety of putative cytochromes and other proteins supporting aerobic respiration and electron transfer were encoded by one or more of *Nab. magadii* replicons. The genome encodes a number of putative proteases/peptidases as well as protein secretion functions. Genes encoding putative transcriptional regulators, basal transcription factors, signal perception/transduction proteins, and chemotaxis/phototaxis proteins were abundant in the genome. Pathways for the biosynthesis of thiamine, riboflavin, heme, cobalamin, coenzyme F_420_ and other essential co-factors were deduced by in depth sequence analyses. However, approximately 36% of *Nab. magadii* protein coding genes could not be assigned a function based on Blast analysis and have been annotated as encoding hypothetical or conserved hypothetical proteins. Furthermore, despite extensive comparative genomic analyses, genes necessary for survival in alkaline conditions could not be identified in *Nab. magadii*.

**Conclusions:**

Based on genomic analyses, *Nab. magadii* is predicted to be metabolically versatile and it could use different carbon and energy sources to sustain growth. *Nab. magadii* has the genetic potential to adapt to its milieu by intracellular accumulation of inorganic cations and/or neutral organic compounds. The identification of *Nab. magadii* genes involved in coenzyme biosynthesis is a necessary step toward further reconstruction of the metabolic pathways in halophilic archaea and other extremophiles. The knowledge gained from the genome sequence of this haloalkaliphilic archaeon is highly valuable in advancing the applications of extremophiles and their enzymes.

## Background

Archaea are the least well-characterized members among the extant three domains of life, and recent genome sequencing efforts have facilitated our understanding of these unusual microbes [1]. The phylum *Euryarchaeota* contains a diverse array of archaea currently classified under eight named classes (*Archaeoglobi, Halobacteria, Methanobacteria, Methanococci, Methanomicrobia, Methanopyri, Thermococci,* and *Thermoplasmata*) and ten orders [2]. Members of the *Euryarchaeota*, particularly those of *Halobacteria*, have received attention because of their ecological and evolutionary importance. Halophilic archaea are physiologically and phylogenetically diverse and occur in a wide variety of environments [3,4]. Most halophilic archaea thrive in hypersaline environments (>15% NaCl). To survive in such extreme conditions, these organisms have evolved strategies to cope with not only osmotic stress and desiccation, but also oxygen limitation and the damaging effects of UV light [5]. The haloalkaliphiles constitute a distinct group of microorganisms since they survive in two extremes: high pH and hypersalinity [6,7]. In addition, haloalkaliphilic archaea have an asymmetric C_20_ C_25_ diether isoprenoid core lipid that is uncommon among neutrophilic halophilic archaea [8].

The genus *Natrialba* within *Halobacteria* is a heterogeneous group of halophiles including those that thrive in neutral as well as alkaline environments [9]. The type species *Natrialba asiatica*, which was isolated from a beach in Japan, is non-alkaliphilic and requires a pH of 6.6 to 7.0 for optimum growth [10]. *Natrialba magadii* (formerly *Natronobacterium magadii*, a strictly aerobic chemoorganotroph isolated from Lake Magadi in Kenya) is an obligately haloalkaliphilic archaeon that requires 20% (3.5 M) NaCl, pH 9.5, and 37 to 40°C for optimum growth [9,11]. In contrast to the white-yellow color of *Nab. asiaticaNab. magadii* is red-orange colored due to the presence of carotenoid pigments in the cell membrane [12]. Furthermore, *Nab. magadii* lacks glycolipids, whereas *Nab. asiatica* contains bis-sulfated glycolipid S_2_-DGD {2, 3-diphytanyl- or phytanyl-sesterterpenyl-1-[2, 6-(HSO_3_)_2_-α-Manp-1 → 2-Glcp]-sn-glycerol} [9,13]. Previous work has demonstrated that *Nab*. *magadii* synthesizes and accumulates 2-sulfotrahalose as an osmolyte under hypersaline conditions [14]. The biochemical features of the flagellar apparatus, a nucleoside diphosphate kinase, a leucine dehydrogenase, and an extracellular serine protease of *Nab. magadi* have also been characterized since its discovery [15-18].

Although knowledge on the biology of halophilic archaea has greatly advanced during the last decade, attempts to understand the physiology and genetics of the haloalkaliphilic archaea are scarce [19]. The study of haloalkaliphilic archaea is interesting from several perspectives because these are among the most alkaliphilic microorganisms reported to date [20]. Of the halophilic archaea related to *Nab. magadii*, a low pass genomic sequence of *Nab. asiatica* strain ATCC 700177 has been reported [21]. The genome of *Haloterrigena turkmenica* strain DSM 5511, a halophilic archaeon isolated from sulfate saline soil in Turkmenistan, is complete and contains 5,287 protein coding genes [22]. Furthermore, the complete genome of *Natronomonas pharaonis* DSM 2160, a haloalkaliphilic archaeon isolated from a soda lake in Egypt, contains 2,843 protein coding genes [23]. In addition, a detailed analysis of the metabolic pathways of halophilic archaea has been described [24]. The goal of the current study was to explore the physiology of *Nab. magadii* at the whole genome level and perform comparative genomic studies with other halophilic as well as haloalkaliphilic archaea. An exploration of the pathways of coenzyme biosynthesis and proteolysis within *Nab. magadii* was also envisaged.

## Methods

*Nab. magadii* was grown at 37°C aerobically in liquid medium containing 20 g/L yeast extract using the method of Tindall *et al*. [11], and genomic DNA was extracted from the pelleted cells using the procedure described by Ng *et al*. for *Halobacterium halobium* (*salinarum*) [25]. Genomic library construction, sequencing, and finishing were performed at the Joint Genome Institute (JGI) facilities at Walnut Creek and the Genome Science facilities at Los Alamos National Laboratory. Briefly, the draft genome of *Nab. magadii* was sequenced using a combination of both Sanger and 454 technologies. A Sanger whole genome shotgun library, which produced 26,484 reads with an average insert size of 6.5 kb, and a 454 FLX standard library, which generated 96.3 Mbp of data, were constructed for this genome. All general aspects of library construction and sequencing performed at the JGI can be found at http://www.jgi.doe.gov/. The Phred/Phrap/Consed software package (http://www.phrap.com) was used for sequence assembly and quality assessment. After the shotgun stage, reads were assembled with parallel phrap (High Performance Software, LLC). Possible mis-assemblies were corrected with Dupfinisher or transposon bombing of bridging clones (Epicentre Biotechnologies, Madison, WI). Gaps between contigs were closed by editing in Consed, custom primer walk, or PCR amplification (Roche Applied Science, Indianapolis, IN). A total of 594 additional custom primer reactions were necessary to close all gaps and raise the quality of the finished sequence. The estimated error rate for the completed genome of *Nab. magadii* was less than 1 in 100,000. The final assembly was based on 19.1 Mbp of Sanger draft data, which provided 4.3x coverage of the genome, and 96.3 Mbp of 454 draft data, which provided 21.7x coverage of the genome.

Preliminary automated annotation, prediction of the number of subsystems, and pairwise BLAST comparisons of protein sets within different strains were performed using the Rapid Annotation using Subsystems Technology (RAST), which is a fully automated, prokaryotic genome annotation service [26]. Subsequently, a detailed manual curation was performed to ensure consistency with the annotation of other halophilic archaea. Annotation of genes involved in coenzyme biosynthesis was based on the information available in recent literature and/or their relatedness to functionally characterized homologs present in other organisms. These annotation details are provided at the web site http://wiki.rzg.mpg.de/HaloferaxWiki. Proteins deemed to be specific to *Nab. magadii* were compared against the NCBI non-redundant protein database to determine whether they were hypothetical or conserved hypothetical. If there was no adequate alignment with any protein (less than 25% identity or aligned region is less than 25% of the predicted protein length), the translated ORF was named a hypothetical protein.

Multiple genome comparisons were performed using the ‘progressive alignment’ option available in the program MAUVE version 2.3.0 [27,28]. Default scoring and parameters were used for generating the alignment. Prior to the alignment, the *Nab. magadii* genome sequence was rearranged to facilitate visual comparison. This was accomplished using the Artemis Comparison Tool to identify a coordinate where the sequence was shifted relative to that of *Htg. turkmenica*. The coordinate was located at 1961610 bp and the *Nab. magadii* sequence was cut starting at this coordinate until the end of the sequence and placed at the beginning of the fasta file so that the genome start was near the major origin of replication.

A synteny plot was generated using the program NUCmer, which uses exact matching, clustering, and alignment extension strategies to create a dot plot based on the number of identical alignments between two genomes [29]. NUCmer was used with the maxmatch argument and, to be consistent with the MAUVE comparison, the rearranged *Nab. magadii* sequence was aligned with that of *Htg. turkmenica*. The *Nab. magadii* genome project is deposited in the Genomes OnLine Database (GOLD) and the complete genome sequence is available from GenBank/EMBL/DDBJ with accession numbers CP001932, CP001933, CP001934, and CP001935. The genome of *Nab*. *magadii* is also accessible through HaloLex (http://www.halolex.mpg.de) and the UCSC Archaeal genome browser (http://archaea.ucsc.edu/).

## Results and discussion

### *Nab. magadii* genome features and comparison with the genomes of other halophilic archaea

The complete genome sequence of *Nab. magadii* consisted of four replicons (total size 4,443,643 bp). Three of these elements had a GC content of ~61% whereas pNMAG02 had a GC content of 56.82%. A comparison of some of the relevant features of these four elements is shown in Table 1. A BLASTN analysis of pNMAG03 on the NCBI database revealed 99% identity to halovirus φCh1 (58,498 bp; GenBank accession number NC_004084), a bacteriophage-like element isolated from *Nab. magadii*. Since halovirus φCh1 has already been described elsewhere [30-32], the analysis of pNMAG03 was excluded from the scope of the current work. The large chromosome of *Nab. magadii* contained two genes encoding putative replication factor C-like proteins (Nmag_1868 and 1910). The large chromosome, pNMAG01, and pNMAG02 were predicted to replicate using a conserved archaeal mechanism [33], since each of these replicons contained at least one gene encoding an Orc1/Cdc6 family replication initiation protein. For the large chromosome, the major replication origin was predicted to be at ca 1.9 Mb, located between Orc1 (Nmag_1930) on the forward strand and a three-gene operon on the reverse strand (Nmag_1927-1929). This set of four highly conserved genes was found adjacent to the replication origin in almost all halophilic archaea.

**Table 1:** **Characteristics of the replicons of*Natrialba magadii*ATCC 43099**

Replicon	Large chromosome	Small chromosome	Large plasmid	Virus φCh1
Annotation	None	pNMAG01	pNMAG02	pNMAG03
Topology	Circular	Circular	Circular	Linear/Circular
Size	3,751,858 bp	378,348 bp	254,950 bp	58,487 bp
GC content	61.42%	60.09%	56.82%	61.90%
Number of RNA genes	53	7	None	None
Number of protein-coding genes	3559 (+1790/-1769)	340 (+183/-157)	219 (+110/-109)	94 (+11/-83)
Number of hypothetical proteins	1278	96	69	75
Full length orc1/cdc6 homologs	5	1	1	None
Glycosyltransferase genes	19	3	1	None
IS elements	21	2	13	None
Overall coding density	83%	80%	76%	93%
GenBank accession number	CP001932	CP001933	CP001934	CP001935

Archaeal genomes can contain a large number of transposable elements and the variety of archaeal insertion sequences is thought to approximate that of bacteria [34]. However, most archaeal genomes lack prophage elements [35]. Manual curation indicated that the genome of *Nab*. *magadii* contained ~36 full-length or truncated genes encoding putative transposases. These insertion sequence elements were scattered throughout the chromosomes and about 20 of these belong to the *IS*605 OrfB family. The *IS*605 OrfB transposase (also called *IS*1341-type transposase) genes were highly diverse, as is typical of halophilic archaea [36]. A single *IS*605 OrfA (Nmag_4105, also called *IS*200-type transposase) was identified in the genome. Other transposase genes in *Nab. magadii* include 7 of the broad category *IS*4 (3 *IS*7-type and 4 *IS*9-type), a single *IS*240-type, and 4 related to *IS*Sod10. The small number of transposase genes and their heterogeneity may indicate that *Nab. magadii* is only minimally affected by these elements. The genome also contained several genes related to bacteriophage elements (*e.g*., PhiH1 repressor protein, phage tail proteins, and phage protein D) and a *vgr*-like gene related to recombination hot spot elements. In addition, there were 13 genes encoding integrase/recombinase-like proteins (Additional file 1: Table S1).

Archaeal genomes generally have 1–4 rRNA operons consisting of the 16 S, 23 S, and 5 S rRNA genes with a tRNA^Ala^ gene located in the internal transcribed spacer [37]. The large chromosome of *Nab*. *magadii* contained two copies of 16 S rRNA-tRNA^Ala^-23 S rRNA-5 S rRNA sequences, one each on the plus and minus strands, as well as two genes encoding components of the RNA guide machinery (Nmag_0693-0694) with fibrillarin-like RNA methyltransferase as the catalytic component. The small chromosome pNMAG01 contained a copy of 16 S rRNA-tRNA^Ala^-23 S rRNA-5 S rRNA sequence on the minus strand and a copy of 23 S rRNA-5 S rRNA sequence on the plus strand. The three 16 S rRNA-tRNA^Ala^-23 S rRNA-5 S rRNA sequences of *Nab*. *magadii* had 99% nucleotide identity to each other. The small chromosome pNMAG01 also contained an orphan 5 S rRNA sequence that had 89% nucleotide identity to the other four 5 S rRNA genes of *Nab. magadii*. Since pNMAG02 lacked rRNA operons and had a lesser GC content than the large and small chromosomes, this self replicating element could be considered a large plasmid. The heterogeneity of the rRNA operons within *Nab*. *magadii* is not a unique feature and the occurrence of such rRNA operons among halobacterial genomes is thought to be due to recombination between rRNA genes of different strains or species [38]. The 16 S rRNA genes of *Nab. magadii* were closely related to those of *Nab. asiatica* (97% identity), *Htg. turkmenica* (96% identity), and *Nmn. pharaonis* (90% identity). Furthermore, the genome of *Nab. magadii* was compared to 17 complete haloarchaeal genomes available in the public databases (Additional file 2: Table S2). Based on this analysis, *Htg. turkmenica* contained the highest number of orthologs (2601 symmetrical hits), followed by *Halopiger xanaduensis* strain SH-6 (2533 symmetrical hits). There were lesser number of orthologs (1805 symmetrical hits) in *Nmn*. *pharaonis*, which has a relatively smaller genome. However, when the data for the percentage of proteins having a bidirectional best blast hit in *Nab. magadii* was computed (Additional file 2: Table S2), *Nmn*. *pharaonis* was the top (63% of the proteins having a bidirectional best blast pair), followed by *Hpg. xanaduensis* (60%) and *Htg. turkmenica* (51%). Results from *Nmn*. *pharaonis* and *Htg. turkmenica* are emphasized in this paper since the former was the only other haloalkaliphilic archaeon with a complete genome sequence and the latter contained the highest number of orthologs.

**Figure 1:**
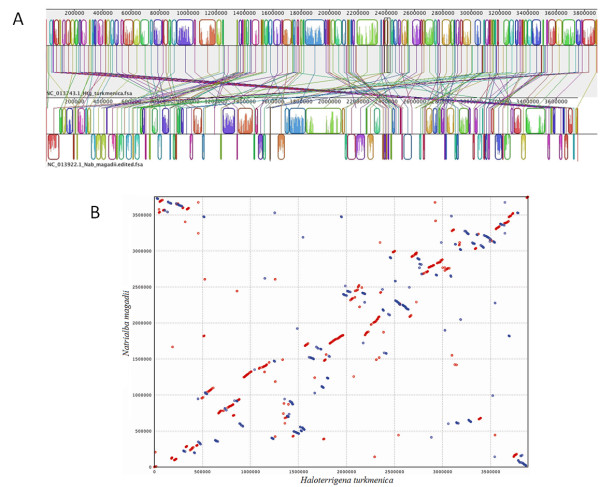
**A. Alignment of the large chromosomes of*Natrialba magadii*ATCC 43099 and*Haloterrigena turkmenica*DSM 5511 using MAUVE 2.** Prior to the alignment, the *Nab. magadii* genome sequence was rearranged to facilitate visual comparison. The *Nab. magadii* sequence was cut starting at 1961610 bp (located between Nmag_1929 and Nmag_1930, encoding putative GTP-binding protein and ORC1 replication initiation protein, respectively) until the end of the sequence and placed at the beginning of the fasta file so that the genome start was near the major origin of replication. Identically colored boxes, known as locally collinear blocks (LCBs), depict homologous regions in the two chromosomes. The edges of LCBs indicate chromosome rearrangements due to recombination, insertions, and/or inversions. Sequences of *Nab. magadii* inverted in relation to those of *Htg. turkmenica* are shown as blocks below the horizontal line. The vertical lines connecting the LCBs point to regions of homology among the two chromosomes. Numbers above the maps indicate nucleotide positions within the respective chromosomes. B. Synteny plot of the large chromosomes of *Natrialba magadii* ATCC 43099 and *Haloterrigena turkmenica* DSM 5511 generated by NUCmer. NUCmer was used with the maxmatch argument and the *Nab. magadii* genome sequence was rearranged as in Figure 1A to facilitate visual comparison. Regions of identity between the two chromosomes were plotted based on pair-wise alignments. Numbers indicate nucleotide positions within the respective chromosomes. Plus strand matches are slanted from the bottom left to the upper right corner and are shown in red. Minus strand matches are slanted from the upper left to the lower right corner and are shown in blue. The number of dots/lines shown in the plot is the same as the number of exact matches found by NUCmer.

The combined size of the complete genome of *Nab*. *magadii* was 1.7 Mb larger than the complete genome of *Nmn*. *pharaonis*, which consists of three replicons (total size 2,749,696 bp). However, *Nab*. *magadii* genome was 1 Mb smaller than the complete genome of *Htg. turkmenica*, which consists of seven replicons (total size 5,440,782 bp). The GC content (61.42%) of the large chromosome of *Nab*. *magadii* was slightly lesser than that of the large chromosomes of *Htg. turkmenica* (~66% GC) and *Nmn*. *pharaonis* (~63% GC). Alignment of the large chromosome of *Nmn*. *pharaonis* (2,595,221 bp) with that of *Nab*. *magadii* using MAUVE showed the presence of very few short syntenic regions (data not shown), whereas a similar alignment using the large chromosome of *Htg. turkmenica* (3,889,038 bp) showed the presence of numerous short syntenic regions (Figure 1A). To further dissect this co-linearity, a BLASTN comparison of the large chromosomes of *Nab*. *magadii* and *Htg. turkmenica* was performed. This analysis revealed the presence of 400 homologous regions (226 plus/plus and 174 plus/minus, sequence range >300 bp, but <2,000 bp) with an average nucleic acid identity of 89% (the identity range was 84%-98%, E-value = 0). The plus and minus strand matches among the chromosomes of *Htg. turkmenica* and *Nab*. *magadii* generated by NUCmer are shown in Figure 1B.

A three-way comparison of all predicted protein-coding genes of *Nab*. *magadii* using the TaxPlot tool of NCBI revealed that *Htg. turkmenica* contained 2387 orthologs, whereas *Nmn*. *pharaonis* contained only 426 orthologs (symmetrical hits). These analyses further confirmed that *Nab*. *magadii* was more closely related to *Htg. turkmenica* than to *Nmn. pharaonis*. In *Nab*. *magadii*, 1518 genes could not be assigned a function based on BLAST analysis and were annotated as encoding hypothetical or conserved hypothetical proteins. The isoelectric point (pI) of most of the predicted proteins of *Nab*. *magadii* was in the 3–5 range, indicating that the general proteome is acidic, which is typical of most halophilic archaea. A two-way comparison of the large chromosomes revealed that *Nab*. *magadii* contained ~945 putative protein-coding genes that had no homologs in *Htg. turkmenica*. A vast majority (~75%) of these *Nab*. *magadii*-specific genes encoded hypothetical proteins. Other genome-specific genes in *Nab*. *magadii* encoded ABC-type transporters, ATPases, kinases, phosphatases, proteases, and oxidoreductases. The genome of *Nab*. *magadii* also contained a variety of simple sequence repeats encoding characteristic peptide repeat patterns.

### General adaptive features

In addition to maintaining an acidic proteome and a cell wall composed of acidic glycoproteins, haloalkaliphilic species appear to have evolved several other mechanisms of adaptation to their niche [39-41]. These include, but are not limited to, intracellular accumulation of inorganic cations and/or neutral organic compounds [42-44]. Halophilic archaea maintain the necessary water balance and osmotic pressure even when the extracellular Na^+^ concentration exceeds 5 M by pumping Na^+^ out and K^+^ into the cell using a variety of cation/proton antiporters [12,45,46]. The genome of *Nab*. *magadii* contained an operon of nine genes encoding a putative pH adaptation K^+^ efflux system (Nmag_3445-3453). Genes related to this operon were present in several halophilic archaea, indicating that they may not encode a specialized system involved in stress response to alkaline growth conditions. Apart from this operon, the genome contained three other genes encoding putative cation/proton antiporters and a gene encoding a putative OsmC family protein (Additional file 1: Table S1).

Low molecular weight organic compounds such as amino acids, polyols, and sugars facilitate cellular adaptation to high-osmolarity and are referred to as osmoprotectants or compatible solutes [47]. Halophilic species also accumulate neutral organic compounds as a means of adaptation to their niche [14,43,44]. The large chromosome of *Nab*. *magadii* contained a locus encoding a putative trehalose-phosphate synthase and a trehalose-phosphatase, which may be involved in the biosynthesis of the osmoprotectant 2-sulfotrehalose. The orthologs of these genes were found in few other halophilic archaea and the osmolyte has been detected by nuclear magnetic resonance spectroscopic analysis in *Nab*. *magadii*[14]. *Nab. magadii* also contained genes encoding the biosynthesis of spermine as well as transporters for the uptake of choline/carnitine/betaine and spermidine/putrescine, which may also provide protection at high-osmolarity (Additional file 1: Table S1). Therefore, it appeared that *Nab. magadii* had multiple mechanisms (*e.g.*, intracellular accumulation of inorganic cations as well as neutral and/or charged organic compounds) for osmotic adaptation.

The scarcity of molecular oxygen in a hypersaline milieu could be a growth-limiting factor for aerobic chemoorganotrophic prokaryotes [48-50]. It has been proposed that some archaeal species accumulate intracellular gas vesicles that help them float on the surface and perform oxidative respiration in their native saturated saltwater habitats [12]. The large chromosome of *Nab*. *magadii* contained a cluster of 11 genes encoding putative gas vesicle synthesis family proteins, which were related to the gas vesicle family proteins of *Hbt. salinarum*. However, *Nab*. *magadii* lacked genes related to those encoding the minor gas vesicle protein (GvpC) and the regulators (GvpD and GvpE). The gas vesicle gene clusters of *Nab*. *magadii* and *Htg. turkmenica* were highly similar to each other and appeared to contain a distant homolog of the *Hbt. salinarum gvpI* gene (Nmag_0338 and Htur_2370, respectively). Nevertheless, these genes encode putative proteins with an N-terminal extension of more than 200 residues not found in GvpI of *Hbt. salinarum*. Furthermore, the gas vesicle clusters of *Nab*. *magadii* and *Htg. turkmenica* contained an additional gene (Nmag_0337 and Htur_2371, respectively) that was absent in *Hbt. salinarum*. *Nab*. *magadii* also contained a gene encoding a hemAT-type aerotactic transducer with a putative globin-coupled sensor protein comprising of a globin fold domain and a methyl-accepting chemotaxis transducer domain (Additional file 1: Table S1). These traits, in addition to the osmotic adaptation mechanisms discussed above, may play a role in the survival of *Nab*. *magadii* in its natural environment.

Other features likely to facilitate the adaptation of *Nab. magadii* to its niche included genes encoding putative mechanosensitive ion channels (MscS) that afford protection against hypoosmotic shock, chaperone proteins DnaJ and DnaK, a thermosome, and heat shock proteins that may participate in protein quality control and cellular response to stress. *Nab*. *magadii* also contained 47 genes (26 on the large chromosome, 18 on pNMAG01, and 3 on pNMAG02) encoding putative proteins of various sizes with a universal stress protein (UspA) domain. One of these genes (Nmag_1302) appeared to form an operon with a gene encoding a putative GCN5-related N-acetyltransferase (GNAT, Nmag_1303), and a similar gene pair was found in *Htg. turkmenica* (Htur_3429-3430, 80% identity, E-value = 2e-82 to 1e-52) and *Nmn*. *pharaonis* (NP1710A-NP1712A, 49% identity, E-value = 2e-41 to 4e-28). It is possible that the GNAT is involved in the acetylation of the linked universal stress protein in these species. In addition, the large chromosome of *Nab*. *magadii* contained genes encoding a superoxide dismutase (*sodA*), two catalases (*katG*, which is common to most halophiles, and *katE*, which is closely related to *katE* of methanogens, also present in *Htg. turkmenica* and *Hpg. xanaduensis*, but not in other halophiles), two alkyl-hydroperoxidase-like proteins, a carbonic anhydrase, and methionine sulfoxide reductases (*msrA* and *msrB*; Additional file 1: Table S1). It is possible that these enzymes have a role in the adaptation of this haloarchaeon to various oxidative stresses associated with energy metabolism.

Furthermore, *Nab*. *magadii* contained genes encoding metal transport proteins and a putative copper resistance protein. *Nab*. *magadii* copper resistance protein appears to contain fused CopC-CopD domains and a distant homolog of this protein occurs in *Nmn. pharaonis* (NP4610A, 39% identity, E-value = 2e-105), but not in other archaea. These genes may be involved in metal homeostasis in the hypersaline environment that *Nab*. *magadii* inhabits. *Nab*. *magadii* also encoded DNA methylases, DNA damage repair excinuclease ABC subunits, DNA mismatch repair proteins, and DNA repair/recombination proteins RadA and RadB (Additional file 1: Table S1). Homologs of these genes are found in several other archaea and they are predicted to be involved in stress response and maintaining genetic integrity.

### Proteases, peptidases, protease inhibitors, and protein translocation

At least 83 genes encoding various types of peptidases/proteases were identified in the genome of *Nab*. *magadii* by manual curation (~1.8% of all protein-coding genes; Additional file 3: Table S3). Interestingly, *Nab. magadii* appears to encode a larger set of proteolytic enzymes compared to most halophilic archaea, including *Nmn. pharaonis*, *Hfx. volcanii* and *Hbt. salinarum*. This suggests that the natural environment inhabited by *Nab. magadii* contains an ample supply of protein debris, which could be used as a major carbon and nitrogen source. The closest homologs of the vast majority of *Nab. magadii* genes (~50%) encoding putative peptidases/proteases were found in *Htg. turkmenica* (Additional file 3: Table S3). Most of the *Nab. magadii* predicted proteases belong to the catalytic type of metallo- and serine proteases. Other proteases include various amino- and carboxypeptidases, oligopeptidases, signal peptidases, ATP-dependent proteases, and intramembrane cleaving proteases (I-CLiPs).

Subtilases (COG1404; subtilisin-like serine proteases) are a large superfamily of functionally diverse endo- and exo-peptidases that occur in prokaryotes and eukaryotes [51]. *Nab*. *magadii* contained nine genes encoding putative S8 and S53 subtilisin kexin sedolisins (Additional file 3: Table S3). Although the predicted subtilisins of *Nab*. *magadii* had diverse sizes (ranging from 402 to 1710 aa), the amino acid motifs containing the catalytic triad (Asp-His-Ser) were conserved in all of them. Six of the predicted subtilisins (Nmag_0073, 0714, 0715, 1249, 1874, and 3633) of *Nab*. *magadii* contained putative targeting signals for translocation through the twin-arginine transport (Tat) pathway, suggesting that these proteases are most likely exported out of the cell. Within this group, Nmag_0715 has been biochemically characterized and designated as the *Natrialba* extracellular protease (Nep) [16]. Nep was demonstrated to be alkali-resistant, a feature that correlates with the conditions that predominate in the natural environment of *Nab*. *magadii*[16]. Interestingly, the C-terminal domain of Nep contains an acidic patch composed of 12 amino acid residues that is absent in the subtilases of neutrophilic organisms [52]. This distinctive feature of Nep may be involved in its stability at high salt and/or high pH. In addition, pNMAG01 contained a gene encoding a putative microcystin LR degradation protein (Nmag_3774, MlrC-like-protein). MlrC peptidases, initially isolated from the bacterium *Sphingomonas,* are a specialized group of metalloproteases assigned to M81 family and they participate in the last step of the degradation pathway of microcystin LR [53]. These enzymes rarely occur in the archaeal domain and the homologs of Nmag_3774 were not found in *Nmn*. *pharaonis* and *Htg. turkmenica* (Additional file 3: Table S3).

All archaeal genomes studied to date are predicted to encode self-compartmentalized proteases (20 S proteasomes and Lon-type proteases) likely to function in energy-dependent proteolysis and an ubiquitin-type mechanism for targeting proteins to proteasomes termed sampylation [54,55]. In archaea, 20 S proteasomes of α- and β-type subunits are thought to function with AAA-ATPases such as the proteasome-activating nucleotidase (PAN) in degrading folded proteins [54]. In addition, ubiquitin-like small archaeal modifier proteins (SAMPs) appear to be conjugated to protein targets by an E1-like enzyme termed ubiquitin-like conjugating enzyme of archaea or UbaA (based on study of *Hfx. volcanii*[56]). The genome of *Nab. magadii* contained an operon encoding putative 20 S proteasome α and β subunits (Nmag_0515-0514, respectively). Apart from this operon, the genome contained separate genes encoding 20 S proteasome α and β subunit homologs (Nmag_3313 and Nmag_3351, respectively). *Nab. magadii* was also predicted to encode homologs of PAN (Nmag_1362 and 2440) and ubiquitin-like small archaeal modifier proteins (SAMPs; Nmag_0567, 1914, 2668, and 2971). The genome of *Nab. magadi* contained two genes encoding putative ubiquitin-like activating enzymes of archaea (UbaA; Nmag_1394 and 3812). Furthermore, it also encoded a distant homolog of UbaA (Nmag_0356) containing a C-terminal JAB1/MPN/Mov34 metalloenzyme (JAMM) domain that was predicted to remove SAMPs from target proteins. In contrast, *Hfx. volcanii* encodes only a single UbaA-type protein that functions in both protein conjugation (sampylation) and sulfur mobilization [56]. *Nab. magadii* also encoded an archaeal-type LonB protease (Nmag_2822), which was demonstrated in its cell membranes [57]. While LonB homologs are conserved and likely act as key energy-dependent proteases in archaea, the physiological significance of these enzymes has not been addressed.

The tetrahedral aminopeptidase (TET protease) is an energy-independent protein complex (with a peptidase domain of the clan MH, family M42, according to the MEROPS database) that was isolated from the neutrophilic haloarchaeon *Har. marismortui*[58]. It has been suggested that TET degrades oligopeptides released by ATP-dependent proteases such as the proteasome and LonB. *Nab. magadii* encodes a homolog of TET (Nmag_1335, peptidase M42 family protein), which, in combination with the energy-dependent proteases, may participate in the intracellular protein turnover in this extremophile. Furthermore, similar to the majority of haloarchaea, *Nab. magadii* appears to encode homologs of the three families of membrane-embedded regulatory proteases denoted as I-CLiPs. These include *sppA*-type signal peptide peptidases (SPPs, Nmag_2612 and 2635), site-2 protease class of zinc metalloproteases that cleave transmembrane domains (S2P type peptidases, Nmag_1508, 1514, 2136, and 3494), and rhomboids (Rho, Nmag_1128, 1636, 2518, and 3579). Furthermore, *Nab. magadii* contained genes encoding type I signal peptidases (sec11-type, Nmag_1326, 1932, 1944, 3375, 3743, and 4175) and a type IV prepilin peptidase (Nmag_1752). The type I signal peptidases and the type IV prepilin peptidase are predicted to be involved in the processing of N-terminal signal peptides of exported proteins and flagellin precursors, respectively.

**Figure 2:**
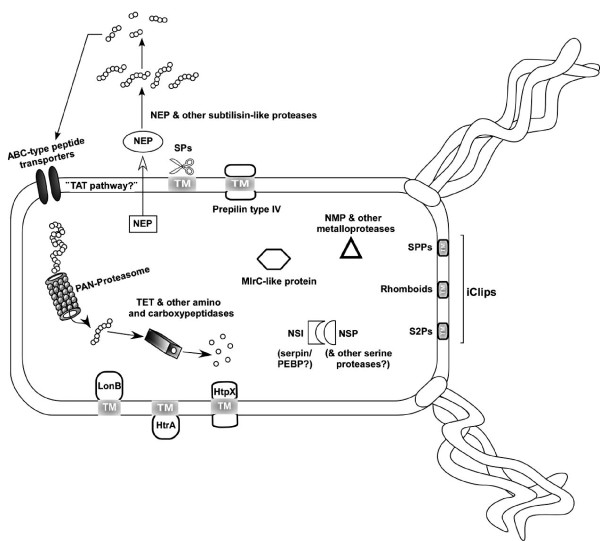
**Schematic representation of*Natrialba magadii*ATCC 43099 proteolytic systems.** The major proteases and protease inhibitor proteins predicted from the analyses of *Nab. magadii* genome are depicted. NSP (*Nab. magadii* serine protease), NMP (*Nab. magadii* metalloprotease), NSI (*Nab. magadii* subtilisin inhibitor), NEP (*Nab. magadii* extracellular protease), and LonB have been described in the literature. SPs, Signal peptidases; I-CLiPs, Intramembrane-Cleaving Proteases; SPPs, Signal peptide peptidases; S2Ps, Site-2 proteases; HtrA, serine protease HtrA; HtpX, putative membrane-bound zinc metalloprotease; TET, Tetrahedral aminopeptidase (Peptidase M42 family); MlrC-like protein, Microcystin LR degradation protein; PEBP, Phosphatidylethanolamine-binding protein (putative protease inhibitor); TM, Transmembrane domain; Tat, Twin arginine translocation pathway.

Cellular protease activity is frequently controlled by endogenous protease inhibitors [59]. Genes encoding putative homologs of protease inhibitors of the serpin (Nmag_2110) and phosphatidylethanolamine-binding protein (Nmag_0329) types were present in *Nab. magadii*. A subtilisin protease inhibitor from this archaeon, denoted NSI, was previously purified and biochemically characterized [60]. This protease inhibitor remains to be investigated at the molecular level and the availability of its gene sequence could facilitate cloning and expression of the recombinant protein for further analysis. A representation of the major proteolytic systems (predicted and/or validated by detecting mRNA and/or assaying protein activity) of *Nab. magadii* is presented in Figure 2. Although this depiction assumes that the proteolytic systems of *Nab. magadii* are independent of each other, their synergistic action *in vivo* cannot be ruled out.

Protein translocation across the cell membrane in prokaryotes is facilitated by at least three mechanisms including the general secretion (Sec) system, the specialized Tat system, and the highly ornate, substrate-specific secretion systems for delivering effector proteins to target sites [61-63]. *Nab*. *magadii* contained genes that encoded putative components of the Sec system (*secYEGDF*, Nmag_0233, 1140, 1564, 2707, and 2708; *srp19*, Nmag_3604; *srp54*, Nmag_1802; *ftsY*, Nmag_0182) and of the Sec-independent Tat protein translocase complex (*tatC*, Nmag_2050-2051; *tatA*, Nmag_3135). While the Tat pathway is commonly used for a small subset of exported proteins in bacteria, it is a dominant export route in halophilic archaea. Many of the exported proteins are subsequently attached to the cell membrane by a lipid anchor and *Nab. magadii* has 119 genes encoding lipid-modified Tat target proteins, as detected by TatLipo analysis [64]. Furthermore, *Nab. magadii* contained genes encoding putative components of a type II secretion system (Nmag_3137-3138) and an archaeosortase (Nmag_2750) for which 17 targets with PGF_CTERM motif were identified [65].

### N-glycosylation, glycosyltransferases, and polysaccharide biosynthesis

N-glycosylation in archaea and eukaryotes uses dolichol phosphate as the lipid base for the assembly of oligosaccharides [66,67]. Glycosyltransferases (GTs) are key components of N-glycosylation in all three domains of life, and the genome of *Nab*. *magadii* contained 23 genes encoding putative GTs. Based on BLASTP analysis on the NCBI database and the presence of conserved domains, these genes were assigned into the GTA (Nmag_0916, 0926, 1132, 1200, 2046, 2620, 2830, 3015, 3273, 3275, 3807, and 4148) and GTB (Nmag_0132, 0135, 0432, 0925, 2541, 3017, 3285, 3431, 3512, 3832, and 3843) superfamilies. One of these genes (Nmag_3015) is in an operon with Nmag_3011 (hexapeptide repeat-containing transferase, 192 aa), Nmag_3012 (aminotransferase, 481 aa), Nmag_3013 (oxidoreductase, 340 aa), and Nmag_3014 (nucleotide sugar dehydrogenase, 544 aa). *Nab*. *magadii* also contained genes encoding a putative oligosaccharyltransferase subunit (Nmag_0927, *aglB* homolog) and a dolichol kinase-like protein (Nmag_1986). Therefore, *Nab*. *magadii* appears to have the genetic potential for N-glycosylation.

**Figure 3:**
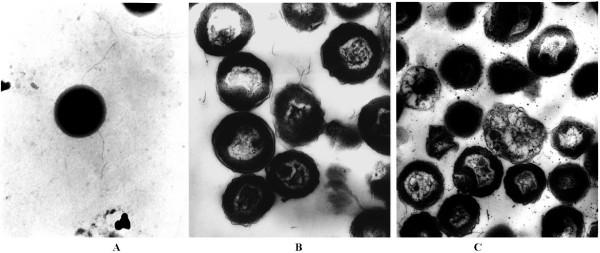
**Transmission electron microscopic images of*Natrialba magadii*ATCC 43099 stationary phase cultures.A**. Negative stain of a single cell with 2% phosphotungstic acid. **B** and **C**. Ultrathin sections stained with uranyl acetate and lead citrate, respectively. The thread shaped appendages in all three images are most likely flagella. The lightly stained material around the cell in panel A is probably an exopolysaccharide.

Several species of halophilic archaea are known to produce copious amounts of extracellular polysaccharides [68]. Although transmission electron microscopic (TEM) images show the presence of an exopolysaccharide-like material around *Nab*. *magadii* cells (Figure 3A), purification and biochemical analyses of this material are yet to be accomplished. *Nab*. *magadii* contained six genes encoding putative polysaccharide biosynthesis proteins (Nmag_0147, 0922, 2457, 3122, 3272, and 3437). Other genes in the genome that encoded putative enzymes involved in polysaccharide biosynthesis included six polysaccharide deacetylases (Nmag_1899, 2045, 2647, 3024, 3271, and 3278), two polyprenyl glycosyl-phosphotransferases (Nmag_0111 and 1184, 65% identity at the predicted protein level), an O-antigen polymerase (Nmag_0143), two UDP-N-acetylglucosamine 2-epimerases (Nmag_0149 and Nmag_0676), an acylneuraminate cytidylyltransferase (Nmag_0148), an O-acetyltransferase (Nmag_0150), a N-acylneuraminate-9-phosphate synthase (Nmag_0151), and two capsule synthesis proteins (Nmag_1511 and 3999). It is possible that some of these genes are involved in the biosynthesis of *Nab*. *magadii* exopolysaccharide- or capsule-like material identified in the TEM images.

### Coenzyme biosynthesis

Archaeal metabolic pathways are unique and diverse, in comparison to those of eubacteria [24]. However, the biosynthesis of several coenzymes involved in archaeal metabolism has only been partially understood. Recent advances in this area include the discovery of a new heme biosynthetic pathway [69], further understanding of the pathway of cobalamin biosynthesis, and the reconstruction of a complete pathway for coenzyme F_420_ biosynthesis in haloarchaea. Analyses of genes putatively involved in coenzyme biosynthesis in *Nab. magadii* was performed in light of these new discoveries. This section describes the genes related to the biosynthesis of thiamine, riboflavin, NAD, coenzyme F_420_, folate, heme, and cobalamin.

Vitamin B_1_ (thiamine pyrophosphate) is involved in several microbial metabolic functions [70]. Prokaryotes have evolved elaborate mechanisms to either synthesize this important co-factor *de novo* or acquire it from their niche [71]. Thiamine biosynthetic pathways among prokaryotes are very diverse [70]. Thiamine biosynthesis is accomplished by joining two intermediate molecules that are synthesized separately. One of these molecules is hydroxymethylpyrimidine pyrophospate (HMP-PP), which is made from aminoimidazole ribotide (AIR, an intermediate of purine biosynthesis) using ThiC and ThiD. The other molecule is hydroxyethylthiazole phosphate (HET-P), which in bacteria is generated by ThiGH and TenI and involves the sulfur carrier ThiS. The sulfur carrier is activated for thiolylation via C-terminal adenylation catalyzed by the N-terminal E1-like domain of ThiI. *Nab. magadii* contained a gene (Nmag_3460) encoding a putative ThiI and several ubiquitin-like β-grasp fold proteins (Nmag_0567, 1914, 2668, and 2971). However, β-grasp fold proteins have multiple functions in halophilic archaea, being involved in sulfur chemistry as well as ubiquitin-like protein modification by SAMPylation [56]. The specific β-grasp fold protein likely to participate in thiamine biosynthesis in *Nab. magadii* remains unidentified.

Furthermore, *Nab. magadii* lacked homologs of ThiG, ThiH, and TenI involved in HET-P biosynthesis in bacteria. Interestingly, *Nab. magadii* encoded a homolog of the yeast HET-P synthase THI4 (Nmag_2419). However, Nmag_2419 is currently annotated as ribose-1,5-bisphosphate isomerase based on the functional characterization of the ortholog MJ0601 from *Methanocaldococcus jannaschii*. In contrast, the ortholog of Nmag_2419 in *Pyrococcus kodokaraensis* (TK0434) does not have ribose-1,5-bisphosphate isomerase activity [72]. Biochemical characterization is required to ascertain the potential role of Nmag_2419 in thiamine biosynthesis. The genome of *Nab. magadii* contained *purM* (Nmag_1281) and *thiC* (Nmag_2593) homologs, which were predicted to be involved in AIR and HMP biosynthesis, respectively (Table 2).

Two distinct proteins, ThiE and ThiN, are known to join HMP-PP and HET-P to generate thiamine phosphate. *Nab. magadii* and other halophilic archaea contain both enzymes. Whereas ThiE (Nmag_1811) is a monofunctional protein, ThiN (Nmag_1282) exists as a C-terminal domain in a ThiDN fusion protein. At the last step, thiamine phosphate is predicted to be further phosphorylated to thiamine pyrophosphate by ThiL (Nmag_1515). Therefore, the conversion of AIR to HMP-PP in *Nab. magadii* appears to be similar to the bacterial pathway and may involve ThiC and ThiD, whereas HET-P biosynthesis in this haloarchaeon appears to be similar to the eukaryotic pathway and may involve Nmag_2419. In addition, *Nab*. *magadii* contained genes encoding a HET kinase (ThiM, Nmag_1810, predicted to be involved in thiamine salvage) and a thiamine transporter (ThiBPQ, Nmag_460-462; ThiB2, Nmag_1940).

Vitamin B_2_ (riboflavin) is the precursor of coenzymes flavin mononucleotide (FMN) and flavin adenine dinucleotide (FAD), which are cofactors for several biochemical reactions [73]. Most bacteria, fungi, and plants can synthesize riboflavin *de novo* using one molecule of GTP and two molecules of ribulose 5-phosphate as substrates [74]. Riboflavin biosynthesis has been described in *M jannaschii*[75]. In general, reduction precedes deamination in the archaeal riboflavin biosynthesis pathway, which appears to be similar to the fungal pathway. In *M. jannaschii*, GTP cyclohydrolase III, the first enzyme of the riboflavin biosynthesis pathway, produces an archaeal-specific formylated intermediate that requires a subsequent deformylation step [75]. However, the haloarchaeal homolog of GTP cyclohydrolase III has not been identified thus far. Furthermore, riboflavin kinases of halophilic archaea are homologous to those of bacteria, but are unrelated to *M. jannaschii* riboflavin kinase. Conversely, riboflavin synthases of halophilic archaea are related to those of *M. jannaschii*, but are unrelated to bacterial riboflavin synthases. Overall, six genes encoding putative enzymes of the riboflavin biosynthesis pathway were identified in *Nab*. *magadii* and only two of these (Nmag_0941 and 0942) were clustered together (Table 2).

Vitamin B_3_ (nicotinic acid) is the central component of coenzymes NAD and NADP, which are essential redox cofactors in metabolism. Like most bacteria, halophilic archaea synthesize NAD from aspartate via quinolinate. *Nab*. *magadii* contained 7 genes (Nmag_2920-2922 form an operon and encode *nadABC*, Nmag_2823 encodes *nadM*, and Nmag_1544, 2475, and 2846 encode *nadE*) that were predicted to be involved in NAD biosynthesis.

Coenzyme F_420_ is involved in methanogenesis [76,77] and other metabolic pathways (*e.g.*, aflatoxin reduction in mycobacteria [78,79]) that require hydride transfer from the low-potential reduced deazaflavin F_420_ to substrates with electron-deficient ring systems. Furthermore, 5-amino-6-(D-ribitylamino)uracil is an intermediate of coenzyme F_420_ and riboflavin biosynthesis pathways [76,77]. Although coenyzme F_420_ has been detected in some halophilic archaea [80], and coenyzme F_420_ biosynthesis genes have been identified among the methanogens [81], its precise function in halophilic archaea is unknown. *Nab*. *magadii* and other halophilic archaea contained several genes encoding putative enzymes of the coenyzme F_420_ biosynthesis pathway. These genes were identified based on the presence of their homologs among the methanogens. Furthermore, using SIMBAL analysis [82], coenyzme F_420_ dependent enzymes have been predicted in halophilic archaea (*e.g.*, NP1902A, TIGRFam 04024, D. Haft, personal communication). *Nab. magadii* predicted proteins with an assigned F_420_-related TIGRFam are shown in Table 2. A possible function of coenzyme F_420_ in the respiratory chain of *Nab*. *magadii* is discussed later in this paper.

Tetrahydrofolate participates in a number of biochemical reactions and reduced folate cofactors are required for the biosynthesis of a variety of molecules in both prokaryotes and eukaryotes [83,84]. The production of folate involves several enzymes catalyzing the pterin and para-aminobenzoic acid branches of the pathway [85]. *Nab. magadii* genes putatively involved in folate biosynthesis were generally similar to those described in *Nmn. pharaonis*, including the archaeal-type GTP cyclohydrolase (Nmag_2853). *Nab. magadii* contained a *pabABC* operon (Nmag_2792-2794) and the homologs of these genes were predicted to be involved in para-aminobenzoate biosynthesis in *Nmn, pharaonis*[24]. *Nab*. *magadii* also contained genes encoding a dihydropteroate synthase, a fused dihydropteroate-dihydrofolate synthase, a dihydrofolate reductase, and a methenyltetrahydrofolate cyclohydrolase (Table 2). The latter enzyme is predicted to participate in the conversion of the C1 metabolite attached to tetrahydrofolate. However, none of the other genes encoding C1-converting enzymes identified in *Haloquadratum* or *Haloarcula* were found in *Nab. magadii* and *Nmn. pharaonis*. Furthermore, *Nab. magadii* contained three genes encoding putative enzymes of the later stages of the folate biosynthesis pathway (FolP, Nmag_0002; FolC-Prd-FolP, Nmag_2554; FolA, Nmag_2988). Nevertheless, genes encoding the bacterial homologs of FolQBK, the atypical FolQ described in *Desulfovibrio*, or the alternative pathway bypassing FolQB (described in *Plasmodium* and several bacteria) were absent in *Nab. magadii* and *Nmn. pharaonis*. Therefore, precise mechanisms of folate biosynthesis in these species remain to be discovered.

Environmental bacteria utilize a variety of redox molecules such as porphyrins and other modified tetrapyrroles like heme, siroheme, and adenosylcobalamin for catalysis, energy transfer, and signal transduction [86]. These tetrapyrroles are synthesized *de novo* using a branched pathway and aminolevulinic acid as the precursor [87,88]. In most prokaryotes, the conversion of glutamate to aminolevulinic acid is catalyzed by glutamyl-tRNA synthetase, glutamyl-tRNA reductase, and glutamate-1-semialdehyde aminotransferase. Two molecules of aminolevulinic acid are condensed by the action porphobilinogen synthase to form porphobilinogen. Four molecules of porphobilinogen are polymerized by the action of the porphobilinogen deaminase to form the tetrapyrrole hydroxymethylbilane. Uroporphyrinogen III methyltransferase cyclizes hydroxymethylbilane to produce uroporphyrinogen III. Uroporphyrinogen III is converted to precorrin-2 in the biosynthetic pathway of adenosylcobalamin and siroheme, which was recently found to be an intermediate of heme biosynthesis (see below).

The complete pathway for the biosynthesis of adenosylcobalamin from precorrin-2 involves two major branches and several enzymes [89,90], some of which are archaea-specific (*e.g.cobYcbiZ*) [91,92]. Halophilic archaea (*e.g.Nmn. pharaonis*) use the “anaerobic” branch, which is characterized by an oxygen-independent ring contraction process (*cibG*) [93]. However, it has been shown that *Halobacterium* synthesizes cobalamin *de novo* under aerobic conditions [91]. The “anaerobic” branch is also characterized by early cobalt insertion and *Nmn. pharaonis* has homologs of the ATP-independent early cobalt chelatase (CbiX) from *Bacillus halodurans* and *Archaeoglobus fulgidus*[94,95]. In the “anaerobic” branch, seven archaeal enzymes are known to be involved in the conversion of precorrin-2 into cobyrinic acid (*cbiLcbiHcbiFcbiGcbiEcbiT*, and *cbiC*), but two pathway gaps (corresponding to *cbiD/cobF* and *cbiJ/cobK*) still remain. A set of 11 genes is known to be involved in conversion of cobyrinic acid into adenosylcobalamin (*cbiAcobA, pduO, cbiPcbiZcbiBcobYcobScobCcobD,* and *cobT*).

Based on genome analyses, it appeared that *Nab*. *magadii* was incapable of *de novo* cobalamin biosynthesis since it lacked the genes encoding enzymes for conversion of precorrin-2 into cobyrinic acid. This is in contrast to *Htg. turkmenica*, which was predicted to be capable of *de novo* cobalamin biosynthesis since it contained the corresponding genes. However, *Nab*. *magadii* was predicted to be capable of corrinoid salvage since it contained a gene encoding a putative corrinoid transporter. *Nab*. *magadii* also contained a set of genes that were predicted to be involved in the conversion of cobyrinic acid into adenosylcobalamin, including a gene (*cbiZ*) that is specific to the archaeal corrinoid salvage pathway (Table 2).

The heme biosynthesis pathway in archaea involving uroporphyrinogen III, precorrin-2, and siroheme appears to be similar to that of *Desulfovibrio*[69]. Conversion of uroporphyrinogen III into siroheme requires three functions (methylation by SirA, iron chelation, and oxidation by SirC). The enzyme catalyzing iron chelation is unknown since the haloarchaeal precorrin-2 dehydrogenase might be monofunctional (as in *Bacillus megaterium* SirC) or might also be a ferrochelatase (as in trifunctional *Escherichia coli * CysG or the bifunctional yeast MET8). From comparison of *Nab*. *magadii* with other halophilic archaea, another possibility emerges: iron chelation may be performed by one of the proteins annotated as CbiX-type cobalt chelatase. *Nmn. pharaonis* has three *cbiX* paralogs (*cbiX1*, NP1108A; *cbiX2*, NP1588A; *cbiX3*, NP0734A), two of which have closely related orthologs in nearly all other halophilic archaea. *Htg. turkmenika*, probably capable of *de novo* cobalamin biosynthesis, has orthologs of both *cbiX1* and *cbiX2*. However, *Nab. magadii*, which is predicted to be incapable of *de novo* cobalamin biosynthesis, and therefore expected to lack these proposed early cobalt chelatases, surprisingly contained a *cbiX2* ortholog (Nmag_3212). It is possible that *cbiX2* functions as a ferrochelatase during siroheme/heme biosynthesis rather than as a cobaltochelatase during *de novo* cobalamin biosynthesis.

Didecarboxysiroheme, a common intermediate of heme and heme *d*1 biosynthesis, is generated by the decarboxylation of siroheme on the C12 and C18 acetyl groups [69]. Siroheme decarboxylase activity is attributed to the *nirDLGH* gene set, which is represented by a pair of two-domain proteins in halophilic archaea (AhbA/NirDL, Nmag_2894; AhbB/NirGH, Nmag_1221). Heme *d*1 is a coenzyme of dissimilatory nitrite reductase (also called cytochrome *cd*_1_) and is not required by organisms lacking this enzyme. The last steps of heme biosynthesis include the removal of acetyl side chains of Fe-coproporphyrin by AhbC (encoded by Mbar_A1793 in *Methanosarcina barkeri*) and the oxidative decarboxylation of heme by AhbD (encoded by DVU_0855 in *Desulfovibrio*) [69] [gene assignments: M. Warren, personal communication, including the information that Supplemental Figure 3 shows the sequence of *ahbC* and not *ahbD*. Orthologs encoding AhbC and AhbD were present in *Nmn. pharaonis* (NP1542A and NP1546A) and *Htg. turkmenica* (Htur_1726 and 1728), but not in *Nab. magadii*. The presence of *ahbC* and *ahbD* in some halophilic archaea but not in others is believed to be due to metabolic heterogeneity rather than incomplete heme biosynthesis. Conversion of heme (also called heme B) into heme A in *Nab. magadii* was predicted to be catalyzed by CtaA and CtaB homologs (*ctaA*, Nmag_0636; *ctaB*, Nmag_2302).

Vitamin H, commonly known as biotin, acts as a coenzyme in several enzyme-catalyzed carboxylation and decarboxylation reactions [96]. Most bacteria can synthesize biotin *de novo* using pimelic acid as a precursor, and some others have evolved mechanisms for importing this essential cofactor from their natural environments [97,98]. Whereas *Nab*. *magadii* is a biotin auxotroph, *Nmn. pharaonis* is a biotin prototroph and the genome of this haloalkaliphilic archaeon has been shown to contain at least three genes putatively involved in the biosynthesis of biotin [24]. The absence of genes for the biosynthesis of biotin in *Nab*. *magadii* was apparent from the analyses of its genome sequence. However, the large chromosome of *Nab. magadii* contained a locus encoding a putative biotin transporter (BioYMN, Nmag_0886-0888), which may facilitate the uptake of biotin from the environment.

### Metabolic and co-factor competency

Nutritional requirements of halophilic archaea in the laboratory are as diverse as their observed phenotypes, suggesting that the metabolic pathways in these organisms are quite intricate [24,99]. The analysis of the genome sequence provided an unprecedented opportunity to comprehend the metabolic versatility of *Nab*. *magadii*. Additional file 4: Table S4 contains a comprehensive list of genes predicted to be involved in a diverse array of functions. Furthermore, genes encoding putative enzymes for archaeal modified pathways of gluconeogenesis and glycolysis as well as those of ribose metabolism and the tricarboxylic acid cycle were present in *Nab*. *magadii*. Genes that encoded putative enzymes for glycerol utilization, aromatic amino acid catabolism, ureagenesis, and urea degradation were also identified in *Nab*. *magadii*. Other putative metabolic features of *Nab*. *magadii* included xylose isomerases, an alpha amylase, a methylglyoxal synthase, sulfatases, a chlorite dismutase, sarcosine oxidases, and aldehyde dehydrogenases (Additional file 4: Table S4).

Molybdenum cofactor (MoCo) is essential for the functioning of molybdoenzymes such as dimethylsulfoxide and trimethylamine-N-oxide reductases, formate dehydrogenases, and nitrate reductases [24,100]. Molybdopterin is the dithiolene-containing tricyclic moiety found within MoCo of all molybdoenzymes except nitrogenases [101]. In bacteria, genes of the *moa, mob, mod, moe*, and *mog* loci have been implicated in the biosynthesis of MoCo using GTP as the substrate [102]. The large chromosome of *Nab*. *magadii* contained 9 genes encoding MoCo biosynthesis functions (Additional file 4: Table S4). It is uncertain if this subset of genes is sufficient for MoCo biosynthesis in *Nab*. *magadii* and biochemical studies are required to test whether this haloarchaeon is molybdenum-dependent.

The haloarchaeon *Haloarcula marismortui* converts acetyl-CoA to glyoxylate via the key intermediate methylaspartate [103]. Glyoxylate is condensed with a second molecule of acetyl-CoA to form malate, which is an intermediate of the tricarboxylic acid cycle. Malate can subsequently be converted to oxaloacetate, which is used by phosphoenolpyruvate carboxykinase for gluconeogenesis. In *Nab. magadii*, activities of the enzymes of the methylaspartate cycle, but not those of the key enzymes of the glyoxylate cycle, were detected [103]. An operon (Nmag_3333-3338) encoding putative homologs of the methylaspartate cycle and a gene encoding a putative phosphoenolpyruvate carboxykinase (Nmag_3507) were present in *Nab*. *magadii*. The square archaeon *Haloquadratum walsbyi* contains a gene (HQ2709A) encoding a phosphoenolpyruvate-dependent phosphotransferase system (PTS) that is predicted to be involved in the phosphorylation of dihydroxyacetone [104]. Homologs of HQ2709A and genes encoding additional PTS components were present in *Htg. turkmenicaHfx. volcanii*, and several other haloarchaeal genomes. However, *Nab. magadii* and *Nmn*. *pharaonis* lacked homologs of these genes encoding PTS components.

### Respiratory chain and ATP synthesis

Running a proton-driven, energy-conserving ATP synthase at high extracellular pH is an obvious challenge. Energy coupling of sodium ions instead of protons was proposed to be an adaptation to alkaliphilic growth conditions and an ATP synthase driven by Na^+^ is the hallmark of such an adaptation. *Nab. magadii* had a cluster of eight genes that form the *atpHIKECFAB* operon encoding putative ATP synthase subunits (Nmag_1370-1377) and an unlinked *atpD* homolog (Nmag_1366). Similar gene clusters were found in several halophilic archaea. Ion specificity of the ATP synthase is determined by the c-ring, which is encoded by the *atpK* gene (Nmag_1375) for A-type ATP synthases. *Nab. magadii* may have a proton-driven ATP synthase since its predicted AtpK lacks the sequence signature of Na^+^-dependent ATP synthases [105]. Instead, within the ion-determining region of AtpK, the sequence (PETLVIL) is identical to that of the proton-driven ATP synthases from *Hfx. volcanii*[106], *Hbt. salinarum*[107], and *Nmn. pharaonis*[23].

Reduction of oxygen and the associated proton-coupled electron transfer (respiration) is the primary source of energy among aerobic organisms. Respiratory complexes, which include a variety of cytochromes and terminal oxidases, are essential components of this process. Biochemical and comparative genomic analyses of the electron transport chain of *Nmn. pharaonis* have revealed several novel features, including a gene encoding a type II NADH dehydrogenase (NP3508A) [23,108]. A homolog of NP3508A in *Acidianus ambivalens* was proposed to be involved in NADH reoxidation, feeding into the lipid-soluble quinone pool [109]. A homolog of NP3508A was also present in *Nab*. *magadii* (Nmag_0301) and several other halophilic archaea. *Nab*. *magadii* also contained genes encoding a putative *nuo* complex (Nmag_3245-3255), which was similar to the mitochrondrial NADH dehydrogenase (complex I). Although 13 *nuo* cluster subunits were conserved among halophilic archaea and *E. coli*, the *nuoEFG* subcomplex, which is involved in accepting NADH, was missing in halophilic archaea [23]. Furthermore, involvement of a type I complex in NADH reoxidation has been ruled out in *Hbt. salinarum*[110]. It is speculated that reduced coenzyme F_420_, which is similar to NADH in its redox potential, may interact with the *nuo* complex in halophilic archaea. In addition to the NADH dehydrogenases, *Nab. magadii* and other halophilic archaea are predicted to encode a succinate dehydrogenase that may oxidize succinate and reduce the quinone pool of the electron transport chain.

A number of cytochromes involved in respiratory electron transport have been characterized among the archaea [12,111]. Terminal oxidases, also known as oxygen reductases, can accept electrons from a variety of donors and reduce dioxygen to water. The large chromosome of *Nab*. *magadii* contained loci encoding putative cytochrome *c*-type terminal oxidase subunits I and II (Nmag_0263-0264) and cytochrome ubiquinol oxidase subunits I and II (Nmag_1036-1037). Furthermore, pNMAG02 contained an operon encoding putative cytochrome ubiquinol oxidase subunits I and II (Nmag_4038-4039) that were related to the proteins encoded by Nmag_1036-1037 (46% identity at the protein level). The homologs of Nmag_0263-0264 and Nmag_1036-1037 were present in *Htg. turkmenica* (Htur_2248- 2249 and Htur_4570- 4571, respectively), but not in *Nmn. pharaonis*. Two *cbaDBAC* operons (plus a monocistronic *cbaE*) encoding putative cytochrome *ba*_3_ terminal oxidase complexes were identified in pNMAG01 (Nmag_3754-3758 and 3802–3805), and these operons appeared to be related to each other (38-65% identity at the predicted protein level). The homologs of these ORFs were present in *Htg. turkmenica* (Htur_0462- 0466) and *Nmn. pharaonis* (NP2960A-NP2968A), the latter of which have been functionally characterized [112]. Halocyanins are predicted to act as one-electron carriers to the terminal oxidases in halophilic archaea [23,108]. This prediction is supported by the observation that *cbaD* is fused to halocyanin in *Hbt. salinarum* and *Har. marismortui*[113]. *Nab*. *magadii* contained several genes encoding putative halocyanin-like proteins (Nmag_2576, 2424, 0446, 0741, 3725, 1878, 3525, 1774, and 3800). Halocyanins are coupled to the reduced quinone pool by the cytochrome *bc*_1_ complex. Although genes encoding a cytochrome *bc*_1_ complex are present in *Hbt. salinarumHfx. volcanii*, and several other halophilic archaea, they were absent in *Htg. turkmenicaNmn. pharaonis*, and *Nab. magadii*. The electron transfer from the reduced lipid-soluble quinone pool to halocyanin remains unresolved in species that lack a cytochrome *bc*_1_ complex. Based on genome comparisons, it appeared that the respiratory chain of *Nab*. *magadii* was more similar to that of *Htg. turkmenica* than to *Nmn. pharaonis*.

Other genes encoding putative cytochrome-related proteins in the large chromosome of *Nab*. *magadii* included Nmag_0636 (cytochrome oxidase assembly protein), Nmag_1972 (cytochrome P450), and Nmag_3057 (cytochrome *c* biogenesis protein). The small chromosome pNMAG01 contained a gene encoding a putative cytochrome *c* biogenesis protein (Nmag_3708) that had 64% identity to the protein encoded by Nmag_3057. *Nab*. *magadii* also contained genes (Nmag_2430-2432) encoding a putative sulfur utilization factor (SUF) system, which was shown to be important for Fe-S cluster biogenesis during stress in *E. coli*[114]. Other genes predicted to participate in bioenergetic conversion in *Nab*. *magadii* include those encoding electron transfer flavoprotein subunits (Nmag_0482-0483 and 1388–1389), SCO1/SenC electron transport proteins (Nmag_0793, 3059, and 3710), and a redoxin domain protein (Nmag_3709).

### Signal transduction, motility, and transcriptional regulation

Two-component signal transduction systems consisting of a histidine kinase (HK) and a response regulator (RR) constitute one of the most frequently encountered bacterial and archaeal communication circuits [115]. *Nab*. *magadii* contained a pair of genes (Nmag_3535-3536) encoding putative HK-RR proteins. *Nab*. *magadii* also contained two pairs of genes (Nmag_2042-2043 and Nmag_3740-3741) encoding putative HK-RR proteins with an additional RR domain in the N-terminus of the predicted HK protein. Furthermore, another set of genes (Nmag_1129-1130) encoding a HK-like protein, which was distantly related to CheA of *Nmn. pharaonis*, and a putative protein containing a RR domain was also predicted in *Nab*. *magadii*. Interestingly, *Nab. magadii* also contained 15 genes encoding putative HK proteins without a cognate RR gene (Nmag_0168, 0323, 0435, 1296, 1909, 2062, 3106, 3188, 3216, 3297, 3954, 4064, 4101, 4114, and 4119) and 11 genes encoding putative RR proteins without a cognate HK gene (Nmag_0951, 1095, 1797, 2147, 2692, 2693, 2877, 3151, 3379, 3389, and 3679).

Halobacterial perception of and response to physical stimulus such as light (phototaxis) is mediated by photoreceptors [116]. The genome of *Nab. magadii* contained a single rhodopsin gene (Nmag_2582, the chloride pump halorhodopsin). *Nab. magadii* also contained 3 genes (Nmag_1701, 2879, and 2881) encoding distant homologs of rhodopsins that were related to each other (36-49% sequence identity). They were also related to NP1758A from *Nmn. pharaonis* (33%-42% sequence identity) and predicted to encode distant rhodopsin homologs that lack the Lys residue involved in covalent retinal attachment. Whether these rhodopsin homologs interact with retinal noncovalently, or if they interact with retinal at all, is unknown. Two of the three genes encoding retinal homologs in *Nab. magadii* (Nmag_2879 and 2881) were located adjacent to genes (Nmag_2880 and 2882) encoding putative methyl-accepting chemotactic transducer proteins. In *Nmn. pharaonis*, NP1758A and NP3132A, which are homologs of Nmag_2879 and 2881, were also found adjacent to genes (NP1756A and NP3134A, respectively) encoding putative methyl-accepting chemotactic transducer proteins [23]. Therefore, this group of distant rhodopsin homologs may be involved in perception of external stimuli, although it remains to be determined if they are involved in light perception. Although *Htg. turkmenica* lacked genes encoding bacteriorhodopsin and halorhodopsin, it contained a single gene (Htur_3663) that appeared to be a distant homolog of Nmag_2879 and 2881. Furthermore, similar to *Nmn. pharaonisNab. magadii* lacked a gene encoding a proton pump bacteriorhodopsin. However, *Nab. magadii* contained a locus encoding putative phytoene desaturase, UbiA prenyltransferase, carotene biosynthesis protein, and phytoene synthase (Nmag_1001-1004) as well as a gene encoding a putative squalene/phytoene synthase (Nmag_2309, unrelated to Nmag_1004).

Two unrelated enzyme families are used for the cleavage of β-carotene into retinal in halophilic archaea. Distant paralogs, which belong to one of these β-carotenase enzyme families, have been identified in *Hbt. salinarum* and designated Brp and Blh [117]. Although homologs of *brp* and *blh* were present in *Nmn. pharaonis* (NP0650A and NP0206A, respectively), they were absent in *Nab. magadii*. However, *Nab. magadii* contained a homolog (Nmag_4083) of *Hqr. walsbyi* HQ2020A, which was predicted to encode a distinct β-carotenase unrelated to those mentioned above [104]. Interestingly, *Htg. turkmenica* lacked homologs of *brp* and *blh* as well as HQ2020A, which is consistent with the absence of all canonical rhodopsins in this organism.

Microbial response to chemical stimulus (chemotaxis, movement toward nutrients or away from stressors) is mediated by chemoreceptors [118]. The large chromosome of *Nab. magadii* contained two loci encoding putative motility and signal transduction functions. One of them contained exclusively “*che* genes” in a *cheYBACCDR* operon (Nmag_3145-3151), which is preceded by two divergently transcribed and distantly related *cheW* genes (Nmag_3152-3153). The *cheYBACCDR* operon encodes a very long signal transduction histidine kinase (CheA, 1576 amino acids), a response regulator receiver protein (CheY), a CheR-type MCP methyltransferase, and a response regulator receiver-modulated methylesterase (CheB). While CheD has been reported to function as glutamine deamidase in some organisms or as methylesterase in others, CheB functions as both [119]. A similar locus was also present in *Htg. turkmenica* (Htur_0954-0962) and based on predicted protein homology, it appeared that the two loci were evolutionarily very closely linked. Highly similar gene clusters were also found in the genomes of *Halopiger xanaduensisNatrinema pellirubrum*, and *Natronobacterium gregoryi*. The second locus (Nmag_2859-2889) contained “*che* genes” along with “*fla* genes” encoding flagellin biosynthesis and assembly functions (see below). The “*che* genes” in this locus encode putative CheA, CheY, CheR, CheB, CheD, a two-domain CheC, and two CheW proteins. *Nab. magadii* contained two *cheF* genes within this locus and homologs of these genes were shown to be involved in chemotaxis in *Halobacterium*[120]. This gene cluster also encoded three methyl-accepting chemotaxis sensory transducers (Nmag_2880, 2882, 2884), two of which were adjacent to genes encoding distant rhodopsin homologs (Nmag_2879 and 2881). Other genes encoding putative methyl-accepting chemotaxis sensory transducers in *Nab. magadii* include Nmag_0478, 0937, 1253, 1386, 1542, 2639, 3325, 3638, and 3856). Among these, two (Nmag_1253, 1386) were adjacent to genes encoding periplasmic ligand-binding proteins.

Archaeal flagella are very different in composition and assembly in comparison to bacterial flagella [121]. In contrast to the bacterial flagellar motor, which is driven by an ion gradient, the archaeal flagellar motor is driven by ATP, as shown in *Hbt. salinarum*[122]. Within the second motility and signal transduction gene cluster (Nmag_2859-2889) of *Nab. magadii* is a region (14,766 bp, 58.66% GC) with 13 predicted ORFs encoding putative flagellin biosynthesis and assembly proteins (Nmag_2862-2874). Except Nmag_2871, which encoded a protein of unknown function, all other ORFs were located on the plus strand. This region contains 4 flagellin genes (Nmag_2862-2865), which encode the flagella proteins previously identified [123] and characterized [17]. Furthermore, *Nab. magadii* contained homologs of *flaF* (Nmag_2866), *flaG* (Nmag_2867), *flaH* (Nmag_2873), *flaI* (Nmag_2874), and *flaJ* (Nmag_2869). The latter two genes (*flaI* and *flaJ*) encode putative proteins homologous to the type II secretion system proteins E and F, respectively. In several archaea, FlaI has been shown to be involved in flagellin assembly [124,125], and was recently proposed as a motor component [126]. The motility gene clusters of halophilic archaea are generally polymorphic, probably due to divergence of genome organization and deletion/duplication of the accessory genes [23]. Nevertheless, *flaHflaI*, and *flaJ* represent a core set of highly conserved genes presumably crucial for archaeal motility.

Since previous electron microscopic analyses have demonstrated that *Nab. magadii* contains distinctive flagella [127-129], and structures resembling flagella are also visible in the TEM images in Figure 3, it is likely that the *fla* locus (Nmag_2862-2874) of *Nab. magadii* was involved in flagellin biosynthesis and motility. In addition, the large chromosome of *Nab. magadii* contained genes encoding a putative full-length PilT protein (Nmag_1543) and a prepilin peptidase (Nmag_1752), whose homologs were found in *Nmn*. *pharaonis* (NP0198A and NP1276A) and *Htg. turkmenica* (Htur_3514 and Htur_0098).

Archaeal basal transcription machinery has many similarities to the eukaryotic RNA polymerase II apparatus. However, the mechanisms of transcription regulation and the transcriptional regulators among archaea are distinct from those of eukaryotes [130,131]. *Nab. magadii* contained 90 genes (~2.2% of all protein-coding genes) encoding putative transcriptional regulators (TRs). BLASTP analyses indicated that most of these predicted proteins were related to bacterial TRs. These TRs were categorized into the following families based on their helix-turn-helix (HTH) motifs (numbers in parenthesis indicate the number of proteins in each family): AsnC (16), IclR (13), PadR (10), ArsR/GntR/Fur (11), XRE (6), TrmB (8), AbrB (3), TetR (3), MarR (3), CopG/Arc/MetJ/NikR (10), HxlR (2), TenA (1), ModE (1), and unassigned (3). Apart from these TRs, *Nab. magadii* also contained 27 genes encoding TRs with an HTH-10 domain, which was also found in bacterio-opsin activators . In addition, *Nab. magadii* encoded a two-domain archaeal histone (Nmag_2205), a single TATA-binding transcription initiation factor (Nmag_1863, 93% protein sequence identity to TbpE from *Hbt. salinarum*), a single transcription initiation factor TFE (Nmag_3157), and a set of seven transcription initiation factors TFB (Nmag_0308, 0527, 2197, 2805, 3037, 3548, and 4179).

## Conclusions

This report describes the genome sequence of *Nab. magadii*, a haloalkaliphilic archaeon that belongs to a physiologically distinct subgroup of halophilic archaea. Although *Nab. magadii* appears to have developed strategies similar to *Nmn. pharaonis* to optimally thrive in low water activity and high pH habitats*,* the genetic architecture of *Nab. magadii* is more similar to that of *Htg. turkmenica* than to *Nmn. pharaonis*. The presence of genes encoding the biosynthesis of the osmoprotectant 2-sulfotrehalose is an uncommon feature among halophilic archaea and this may have contributed to the evolution of *Nab. magadii* in its natural environment. *Nab. magadii* has genes encoding a number of cation/proton antiporters as well as pathways for the biosynthesis and/or transport of various cofactors and vitamins. The occurrence of genes encoding enzymes involved in glycolysis, gluconeogenesis, and glycerol utilization suggests that *Nab. magadii* is metabolically versatile and can use different carbon and energy sources to sustain growth. Furthermore, the large repertoire of genes encoding putative proteases/peptidases and peptide transport systems is indicative of the protein/peptide catabolic potential of *Nab. magadii*. It also appears that *Nab. magadii* can perceive and process physical and chemical stimuli, and respond appropriately by moving toward or away from those stimuli using the flagellar apparatus. The information obtained from this comparative genomic analysis contributes to our overall understanding of the biology and diversity of halophilic archaea. In particular, it will guide current and future research on the genetics and physiology of *Nab. magadii*. Such studies are expected to facilitate the manipulation of this archaeon as a model for haloalkalphilic metabolism and its optimization for biotechnological applications.

## Competing interests

The author(s) declare that they have no competing interests.

## Authors’ contributions

SS performed the annotation using RAST, planned the comparative analysis, and drafted most of the manuscript. JFC contributed to whole genome comparisons and generated Figure 1. JCD, KWD, LAG, NK, RT, SP, SL, and TW contributed to project planning, project management, genome sequencing, and genome annotation. DES and MIG performed the manual annotation of proteolytic enzymes and protease inhibitors and generated the schematic representation of *Nab. magadii* proteases/inhibitors (Figure 2). RAP prepared *Nab. magadii* samples used for TEM analysis. FP participated in the manual curation of *Nab. magadii* genome, reconstructed several coenzyme biosynthetic pathways, and helped draft the manuscript. RDC and JAM conceived the study, participated in genome analyses, and helped draft the manuscript. All authors read and approved the final manuscript.

## Supplementary Material

Additional file 1 **Table S1.***Natrialba magadii* ATCC 43099 genes discussed in the text. This table lists *Nab. magadii* ATCC 43099 genes related to bacteriophage and recombination elements, rRNA genes, and genes encoding adaptive features. Click here for file

Additional file 2 **Table S2. Bidirectional best blast pairs among proteins from***Natrialba magadii* and 17 other haloarchaeal genomes. This table lists the number of bidirectional best blast pairs among proteins from *Nab. magadii* and 17 other halophilic archaea. The first column is the number of total proteins in each genome, the second column is the number of bidirectional best blast pairs, and the third column is the percentage of proteins having a bidirectional best blast hit in *Nab. magadii*. Click here for file

Additional file 3 **Table S3.***Natrialba magadii* ATCC 43099 genes encoding putative peptidases/proteases, protease inhibitors, and regulatory proteins. This table lists *Nab. magadii* ATCC 43099 genes encoding various types of proteases and peptidases as well as protease inhibitors and regulatory proteins. Click here for file

Additional file 4 **Table S4. ***Natrialba magadii* ATCC 43099 genes involved in metabolism. This table lists *Nab. magadii* ATCC 43099 genes encoding molybdenum cofactor biosynthesis and other metabolic functions. Click here for file
