# Supplementary material for: A comparative genomics perspective on the genetic content of the alkaliphilic haloarchaeon Natrialba magadii ATCC 43099T
Source: BMC Genomics. 2012 May 4;13:165. doi: 10.1186/1471-2164-13-165 (PMC3403918; doi:10.1186/1471-2164-13-165)
Supplement: Additional file 1 — Table S1.Natrialba magadii ATCC 43099 genes discussed in the text. This table lists Nab. magadii ATCC 43099 genes related to bacteriophage and recombination elements, rRNA genes, and genes encoding adaptive features. [file 1471-2164-13-165-S1.doc]

| SUPPLEMENTAL TABLE S1: *Natrialba magadii* ATCC 43099 genes related to bacteriophage and recombination elements, rRNA genes and adaptive features. | |
| --- | --- |
| **Related to bacteriophage and recombination elements:** | |
| **Locus tag** | **Annotation** |
| Nmag_0153, 0294, 3625, 3692, 4173, and 4298 | PhiH1 repressor proteins |
| Nmag_0764 | Phage tail protein |
| Nmag_0770 | Phage protein D |
| Nmag_0769 | *vgr* related to hot spot elements |
| Nmag_0046, 0235, 0251, 0295, 0353, 0465, 1605, 3225, 3592, 3790, 4009, 4284, and 4294 | Integrase/recombinase proteins |
| **Related to ribosomal RNA:** | |
| Nmag_R0001-R0004, Nmag_R0023-R0026, and Nmag_R0057-R0060 | 16S rRNA-tRNA^Ala^-23S rRNA-5S rRNA operon |
| Nmag_R0054-R0055 | 23S rRNA-5S rRNA operon |
| Nmag_R0056 | Orphan 5S rRNA |
| Nmag_0693 | RNA methyltransferase complex (Nop5) |
| Nmag_0694 | Fibrillarin-like RNA methyltransferase |
| **General adaptive features:** | |
| Nmag_3445-3453 | pH adaptation K^+^ efflux system operon |
| Nmag_1182, 2706, and 2782 | Cation/proton antiporters |
| Nmag_0879 | Osmotically inducible protein (OsmC) |
| Nmag_3061 | Trehalose-phosphate synthase |
| Nmag_3062 | Trehalose phosphatase |
| Nmag_0842 | Biosynthesis of spermine |
| Nmag_0066 | Choline/carnitine/betaine transporter |
| Nmag_0081-0084, 0096, 0100-0103, 0104, 0106, and 3861-3864 | ABC-type spermidine/putrescine transporter |
| Nmag_0332-0342 | Gas vesicle biosynthesis proteins |
| Nmag_2882 | Aerotaxis transducer (HemAT) |
| Nmag_1302 | UspA domain protein |
| Nmag_1303 | GCN5-related N-acetyltransferase |
| Nmag_0015, 0444, 0505, 0661, and 1093 | Mechanosensitive ion channels (MscS) |
| Nmag_0570 | Chaperone protein (DnaJ) |
| Nmag_0571 | Chaperone protein (DnaK) |
| Nmag_1469, 2095, and 2827 | Thermosome |
| Nmag_0593, 1212, 1704, 2140, and 2767 | Heat shock proteins (Hsp20) |
| Nmag_0456 | Superoxide dismutase (SodA) |
| Nmag_3120 (KatG) | Catalase |
| Nmag_3391 (KatE) | Catalase |
| Nmag_2532 and 2663 | Alkyl-hydroperoxidase-like proteins |
| Nmag_2131 | Carbonic anhydrase |
| Nmag_0346, 1007, and 3077 | Methionine sulfoxide reductases |
| Nmag_0206, 0399, 1348, 1856, 3114, 3706, and 3759 | Metal transport proteins |
| Nmag_0946 | Putative copper resistance protein  (CopCD) |
| Nmag_0041, 0735, 1172, 1620, 4235, and 4246 | DNA methylases |
| Nmag_1263, 2070, and 2078 | DNA damage repair excinuclease ABC subunits |
| Nmag_0330, 1162, 1284, 1289, and 2063 | DNA mismatch repair proteins |
| Nmag_1831 and 0077 | DNA repair/recombination proteins |
