# Supplementary material for: A comparative genomics perspective on the genetic content of the alkaliphilic haloarchaeon Natrialba magadii ATCC 43099T
Source: BMC Genomics. 2012 May 4;13:165. doi: 10.1186/1471-2164-13-165 (PMC3403918; doi:10.1186/1471-2164-13-165)
Supplement: Additional file 2 — Table S2. Bidirectional best blast pairs among proteins fromNatrialba magadii and 17 other haloarchaeal genomes. This table lists the number of bidirectional best blast pairs among proteins from Nab. magadii and 17 other halophilic archaea. The first column is the number of total proteins in each genome, the second column is the number of bidirectional best blast pairs, and the third column is the percentage of proteins having a bidirectional best blast hit in Nab. magadii. [file 1471-2164-13-165-S2.doc]

| SUPPLEMENTAL TABLE S2: Bidirectional best blast pairs among proteins from *Natrialba magadii* and 17 other haloarchaeal genomes | | | |
| --- | --- | --- | --- |
| Total  proteins | Number of bidirectional  best blast pairs | Fraction of proteins having  a bidirectional best blast pair | Species |
| 5113 | 2601 | 50.87 | *Haloterrigena turkmenica* DSM_5511 |
| 4221 | 2533 | 60.01 | *Halopiger xanaduensis* SH-6 |
| 4443 | 2146 | 48.30 | *Haladaptatus paucihalophilus* DX253 |
| 3898 | 2059 | 52.82 | *Halogeometricum borinquense* DSM_11551 |
| 4291 | 2022 | 47.12 | *Haloarcula marismortui* ATCC_43049 |
| 4066 | 2014 | 49.53 | *Haloferax volcanii* DS2 |
| 3870 | 2007 | 51.86 | *Halalkalicoccus jeotgali* B3 |
| 3859 | 1990 | 51.57 | *Haloarcula hispanica* ATCC_33960 |
| 3558 | 1915 | 53.82 | *Halorubrum lacusprofundi* ATCC_49239 |
| 3349 | 1856 | 55.42 | *Halomicrobium mukohataei* DSM_12286 |
| 2852 | 1805 | 63.29 | *Natronomonas pharaonis* DSM_2160 |
| 2998 | 1625 | 54.20 | *Halorhabdus utahensis* DSM_12940 |
| 2832 | 1604 | 56.64 | *Halobacterium salinarum* R1 |
| 3980 | 1601 | 40.23 | *Halorhabdus tiamatea* SARL4B |
| 2987 | 1569 | 52.53 | *Haloquadratum walsbyi* DSM_16854 |
| 2859 | 1553 | 54.32 | *Haloquadratum walsbyi* DSM_16790 |
| 2622 | 1540 | 58.73 | *Halobacterium salinarum* NRC-1 |
