# Supplementary material for: A comparative genomics perspective on the genetic content of the alkaliphilic haloarchaeon Natrialba magadii ATCC 43099T
Source: BMC Genomics. 2012 May 4;13:165. doi: 10.1186/1471-2164-13-165 (PMC3403918; doi:10.1186/1471-2164-13-165)
Supplement: Additional file 3 — Table S3.Natrialba magadii ATCC 43099 genes encoding putative peptidases/proteases, protease inhibitors, and regulatory proteins. This table lists Nab. magadii ATCC 43099 genes encoding various types of proteases and peptidases as well as protease inhibitors and regulatory proteins. [file 1471-2164-13-165-S3.doc]

| SUPPLEMENTAL TABLE S3: *Natrialba magadii* ATCC 43099 genes encoding putative peptidases/proteases, protease inhibitors, and regulatory proteins | | |
| --- | --- | --- |
| Locus tag, Protein, pI,  (Asp + Glu)/ (Arg + Lys) | Annotation | Closest homolog  (locus tag, protein, identity^1^) |
| Nmag_0073, 402 aa, 3.98, 71/17 | Peptidase S8 and S53 subtilisin kexin sedolisin | Htur_2098, 426 aa, 43% |
| Nmag_0329, 220 aa, 6.23, 34/32 | Phosphatidylethanolamine-binding protein (PEBP, putative protease inhibitor) pseudogene | Htur_2383, 153 aa, 62% |
| Nmag_0356, 771 aa, 4.56, 136/65 | UbaA (E1/MoeB/ThiF family) homolog with C-terminal JAMM domain | MXAN_7285, 598 aa, 33% |
| Nmag_0514, 250 aa, 4.57, 39/20 | 20S proteasome, β-type subunit | Huta_2664, 234 aa, 48% |
| Nmag_0515, 280 aa, 5.28, 43/32 | 20S proteasome, α-type subunit | NP3738A, 255 aa, 48% |
| Nmag_0561, 668 aa, 4.01, 151/44 | Peptidase S9 prolyl oligopeptidase | rrnAC2119, 635 aa, 65% |
| Nmag_0567, 88 aa, 4.48, 18/8 | SAMP ubiquitin-like β-grasp fold protein | HacjB3_04780, 90 aa, 55% |
| Nmag_0637, 384 aa, 4.38, 75/34 | Peptidase M24 family | Htur_2092, 367 aa, 73% |
| Nmag_0714, 683 aa, 3.87, 140/34 | Peptidase S8 and S53 subtilisin kexin sedolisin | Htur_2052, 540 aa, 44% |
| Nmag_0715, 541 aa, 3.77, 108/20 | Peptidase S8 and S53 subtilisin kexin sedolisin (Nep) | BAA01049, 530 aa, 76% |
| Nmag_0968, 326 aa, 5.35, 39/ 30 | Peptidase M48 family Ste24p (HtpX) | Nhal_0225, 300 aa, 33% |
| Nmag_1128, 319 aa, 8.92, 14/19 | Rhomboid family protein | Htur_2632, 310 aa, 74% |
| Nmag_1143, 348 aa, 3.53, 75/9 | Peptidase S1 and S6 chymotrypsin/Hap (HtrA) | Htur_2644, 395 aa, 61% |
| Nmag_1249, 431 aa, 5.88, 54/45 | Peptidase S8 and S53 subtilisin kexin sedolisin | EEJ56263, 594 aa, 41% |
| Nmag_1273, 557 aa, 4.95, 94/60 | Metalloendopeptidase, glycoprotease family | Htur_3053, 578 aa, 79% |
| Nmag_1274, 231 aa, 4.15, 41/13 | Pyrrolidone-carboxylate peptidase | CKO_02452, 214 aa, 44% |
| Nmag_1286, 295 aa, 5.19, 26/19 | Peptidase M48 family | Htur_0206, 293 aa, 42% |
| Nmag_1326, 338 aa, 4.04, 48/11 | S26B signal peptidase | HQ1595A, 384 aa, 48% |
| Nmag_1335, 356 aa, 4.16, 66/23 | Peptidase M42 family (TET) | Huta_0675, 359 aa, 58% |
| Nmag_1360, 454 aa, 4.46, 80/35 | Peptidase M28 family | Htur_3368, 459 aa, 69% |
| Nmag_1361, 613 aa, 4.15, 143/46 | Oligoendopeptidase F | Htur_3366, 606 aa, 84% |
| Nmag_1362, 410 aa, 4.40, 94/16 | 26S proteasome subunit P45 family | Htur_3362, 410 aa, 86% |
| Nmag_1394, 278 aa, 4.25, 50/21 | Ubiquitin-like activating enzymes of archaea | Halxa_1982, 279 aa, 87% |
| Nmag_1398, 438 aa, 4.65, 76/47 | Peptidase M24 family | Htur_3264, 410 aa, 54% |
| Nmag_1446, 610 aa, 5.16, 100/67 | Oligoendopeptidase F | Hmuk_0031, 594 aa, 37% |
| Nmag_1508, 612 aa, 4.00, 77/22 | Peptidase M50 family. (S2P) | Htur_3477, 607 aa, 68% |
| Nmag_1514, 392 aa, 4.87, 37/20 | Peptidase M50 family (S2P) | Htur_3483, 388 aa, 66% |
| Nmag_1537, 509 aa, 4.38, 107/38 | Carboxypeptidase M32 family | Hmuk_0281, 509 aa, 56% |
| Nmag_1538, 525 aa, 4.19, 126/41 | Carboxypeptidase M32 family | Htur_3535, 524 aa, 78% |
| Nmag_1552, 184 aa, 6.42, 21/20 | Zinc-dependent peptidase M54 family | Htur_3502, 173 aa, 91% |
| Nmag_1636, 216 aa, 5.15, 14/7 | Rhomboid family protein | Hbor_34800, 199 aa, 40% |
| Nmag_1698, 999 aa, 4.12, 192/55 | Carboxypeptidase A, M14 family | HacjB3_16606, 907 aa, 35% |
| Nmag_1722, 762 aa, 4.06, 164/50 | S9 prolyl oligopeptidase | Htur_0116, 726 aa, 65% |
| Nmag_1752, 352 aa, 4.51, 38/21 | Peptidase A24A prepilin type IV | Htur_0098, 349 aa, 60% |
| Nmag_1777, 380 aa, 4.28, 79/27 | Peptidase M29 aminopeptidase II | Htur_0278, 367 aa, 84% |
| Nmag_1859, 325 aa, 4.69, 47/25 | Peptidase M48 family (HtpX) | Htur_0403, 350 aa, 45% |
| Nmag_1874, 474 aa, 4.05, 82/23 | Peptidase S8 and S53 subtilisin kexin sedolisin | Htur_0058, 449 aa, 67% |
| Nmag_1914, 87 aa, 4.49, 19/10 | Ubiquitin-like small archaeal modifier protein | Htur_0025, 78 aa. 72% |
| Nmag_1932, 353 aa, 4.44, 76/31 | Peptidase S24/S26A/S26B | Htur_0003, 319 aa, 64% |
| Nmag_1944, 288 aa, 4.29, 43/19 | S26B signal peptidase I | Htur_0003, 319 aa, 58% |
| Nmag_1984, 508 aa, 4.09, 97/30 | D-alanyl-D-alanine carboxypeptidase | Snas_1386, 521 aa, 30% |
| Nmag_2035, 297 aa, 6.99, 28/28 | Peptidase M48 family | Htur_0206, 293 aa, 83% |
| Nmag_2136, 415 aa, 4.74, 40/23 | Peptidase M50 family (S2P) | Htur_0289, 393 aa, 70% |
| Nmag_2110, 500 aa, 3.84, 114/23 | Proteinase inhibitor I4 serpin | Htur_0395, 475 aa, 64% |
| Nmag_2142, 320 aa, 4.01, 65/17 | Aminopeptidase M29 family | Htur_0343, 318 aa, 82% |
| Nmag_2255, 388 aa, 4.25, 86/33 | Peptidase M29 aminopeptidase II | Htur_0432, 360 aa, 84% |
| Nmag_2391, 396 aa, 4.24, 80/26 | Peptidase M24 | Htur_1427, 396 aa, 77% |
| Nmag_2440, 405 aa, 4.41, 90/45 | 26S proteasome subunit P45 family | Htur_1492, 405 aa, 92% |
| Nmag_2471, 1489 aa, 3.40, 375/28 | Peptidase S8 and S53 subtilisin kexin sedolisin | Hlac_1628, 1215 aa, 37% |
| Nmag_2505, 424 aa, 4.94, 54/41 | Peptidase M48 family | Huta_1947, 370 aa, 34% |
| Nmag_2518, 225 aa, 10.19, 8/11 | Rhomboid family protein | Halxa_3822, 233 aa, 61% |
| Nmag_2538, 611 aa, 4.06, 129/39 | S9 prolyl oligopeptidase | Htur_3188, 612 aa, 86% |
| Nmag_2612, 334 aa, 4.17, 62/27 | Signal peptide peptidase SppA, 36K type (SPP) | Htur_1351,331 aa,76% |
| Nmag_2635, 328 aa, 3.95, 55/14 | S49 Peptidase (Spp-like homolog) | HVO_1987, 299 aa, 51% |
| Nmag_2668, 111 aa, 4.09, 21/8 | Ubiquitin-like small archaeal modifier protein | Htur_0699, 96 aa, 78% |
| Nmag_2702, 366 aa, 4.37, 56/23 | Peptidase S1 and S6 chymotrypsin (HtrA) | Htur_0739, 371 aa, 65% |
| Nmag_2745, 269 aa, 6.23, 30/28 | Peptidase M48 family (HtpX) | Htur_0795, 274 aa, 67% |
| Nmag_2766, 431 aa, 8.60, 41/43 | Peptidase M48 family | Hlac_2574, 290 aa, 37% |
| Nmag_2822, 778 aa, 4.42, 149/75 | Sigma 54 interacting domain protein  (putative protease S16 family, Lon) | Htur_0860, 731 aa, 82.8% |
| Nmag_2941, 316 aa, 4.38, 54/22 | Beta-aspartyl-peptidase (asparaginase 2) T2 family | HacjB3_06535, 300 aa, 61% |
| Nmag_ 2942, 299 aa, 4.34, 65/23 | Methionine aminopeptidase, type II, M24 family | Htur_0549, 300 aa, 90% |
| Nmag_2952, 394 aa, 5.20, 49/38 | Peptidase M48 family | Huta_1947, 370 aa, 58% |
| Nmag_2971, 94 aa, 3.91, 24/6 | Ubiquitin-like small archaeal modifier protein | Htur_0580, 93 aa, 85% |
| Nmag_2982, 704 aa, 4.23, 137/47 | Prolyl oligopeptidase S9A family | Sthe_2440, 684 aa, 42% |
| Nmag_3113, 398 aa, 5.00, 51/39 | Peptidase M48 family | Huta_1947, 370 aa, 36% |
| Nmag_3159, 420 aa, 4.33, 78/ 32 | Dipeptidyl aminopeptidase S9 family | Namu_5182, 423 aa, 47% |
| Nmag_3241, 436 aa, 4.39, 89/33 | Peptidase M28 family | Htur_1093, 454 aa, 79% |
| Nmag_3313, 251 aa, 4.25, 52/19 | Proteasome endopeptidase complex, alpha subunit | Htur_1707, 256 aa, 91% |
| Nmag_3351, 243 aa, 4.42, 39/16 | Proteasome endopeptidase complex, beta subunit | Htur_1568, 243 aa, 89% |
| Nmag_3375, 397 aa, 4.50, 49/24 | S26B signal peptidase I | Hlac_0480, 387 aa, 41% |
| Nmag_3494, 209 aa, 6.20, 13/10 | Peptidase M50 family (S2P) | Htur_1624, 212 aa, 81% |
| Nmag_3497, 360 aa, 4.19, 73/28 | LD-carboxypeptidase A, S66 family | rrnAC1893, 353 aa, 59% |
| Nmag_3579, 671 aa, 4.72, 83/42 | Rhomboid family protein | Halxa_3310, 622 aa, 55% |
| Nmag_3633, 414 aa, 4.35, 62/25 | Peptidase S8 and S53 subtilisin kexin sedolisin | Htur_2098, 426 aa, 46% |
| Nmag_3653, 457 aa, 4.34, 75/32 | D-alanyl-D-alanine carboxypeptidase S13 family | SULAZ_0570, 456 aa, 30% |
| Nmag_3657, 385 aa, 3.98, 81/27 | NLP/P60 protein, putative peptidase | GFO_3330, 407 aa, 28% |
| Nmag_3662, 621 aa, 4.32, 118/50 | S9 prolyl oligopeptidase | Hlac_0581, 628 aa, 51% |
| Nmag_3743, 385 aa, 4.81, 39/21 | S26B signal peptidase | Htur_3509, 391 aa, 60% |
| Nmag_3774, 510 aa, 4.63, 84/45 | Microcystin LR degradation protein (MlrC-like protein), M81 family | Sthe_1234, 503 aa, 40% |
| Nmag_3812, 278 aa, 4.45, 45/23 | Ubiquitin-like activating enzymes of archaea | Hlac_0421, 270 aa, 55% |
| Nmag_3901, 429 aa, 4.64, 73/38 | M24 family metallopeptidase | Htur_2880, 424 aa, 85% |
| Nmag_3913, 1710 aa, 3.28, 455/23 | Peptidase S8 and S53 subtilisin kexin sedolisin | Hlac_1628, 1215 aa, 36% |
| Nmag_3933, 496 aa, 4.18, 98/32 | Peptidase M20 family | Sthe_2747, 457 aa, 43% |
| Nmag_3953, 378 aa, 4.32, 78/31 | Peptidase M24 family | HVO_A0376, 367 aa, 62% |
| Nmag_3971, 671 aa, 4.61, 115/61 | S9 prolyl oligopeptidase | Acid_1826, 667 aa, 26% |
| Nmag_3990, 447 aa, 4.44, 84/36 | Peptidase M28 family | Htur_1093, 454 aa, 44% |
| Nmag_3997, 456 aa, 4.42, 87/37 | Peptidase M28 family | Hmuk_0415, 440 aa, 47% |
| Nmag_3998, 440 aa, 4.72, 74/ 38 | Peptidase M28 family | Hmuk_0415, 440 aa, 48% |
| Nmag_4003, 279 aa, 4.51, 46/22 | Peptidase M55 (D-aminopeptidase) | BBR47_28010, 278 aa, 40% |
| Nmag_4051, 400 aa, 4.29, 77/24 | Peptidase M24 family | HacjB3_12765, 399 aa, 56% |
| Nmag_4052, 389 aa, 4.62, 70/35 | Peptidase M24 family | HacjB3_13510, 377 aa, 50% |
| Nmag_4175, 372 aa, 4.45, 49/25 | S26B signal peptidase | Hlac_0480, 387 aa, 45% |
| Nmag_4185, 655 aa, 4.35, 123/52 | S9 prolyl oligopeptidase | Sthe_1357, 652 aa, 53% |
| Nmag_4209, 1618 aa, 3.54, 404/31 | Peptidase S8 and S53 subtilisin kexin sedolisin | Hlac_1628, 1215 aa, 42% |
