# Supplementary material for: A comparative genomics perspective on the genetic content of the alkaliphilic haloarchaeon Natrialba magadii ATCC 43099T
Source: BMC Genomics. 2012 May 4;13:165. doi: 10.1186/1471-2164-13-165 (PMC3403918; doi:10.1186/1471-2164-13-165)
Supplement: Additional file 4 — Table S4. Natrialba magadii ATCC 43099 genes involved in metabolism. This table lists Nab. magadii ATCC 43099 genes encoding molybdenum cofactor biosynthesis and other metabolic functions. [file 1471-2164-13-165-S4.doc]

| SUPPLEMENTAL TABLE S4: *Natrialba magadii* ATCC 43099 genes involved in metabolism | | |
| --- | --- | --- |
| Locus tag, Protein, pI,  (Asp + Glu)/ (Arg + Lys) | Annotation | Closest Htur homolog*  (locus tag, protein, identity) |
| **Molybdenum cofactor biosynthesis** | | |
| Nmag_0416, 341 aa, 4.31, 76/32 | Molybdenum cofactor biosynthesis protein MoaA | Htur_2557, 330 aa, 88% |
| Nmag_0517, 182 aa, 4.27, 39/17 | Molybdenum cofactor biosynthesis protein MoaC | Htur_2451, 181 aa, 82% |
| Nmag_0725, 251 aa, 4.13, 49/18 | Molybdopterin binding domain protein | Htur_1918, 248 aa, 75% |
| Nmag_1213, 443 aa, 4.62, 77/40 | Molybdenum cofactor biosynthesis protein MoeA | Htur_3099, 449 aa, 75% |
| Nmag_1214, 682 aa, 4.36, 128/63 | Molybdenum cofactor biosynthesis protein MoeA | Htur_3237, 623 aa, 77% |
| Nmag_1512, 312 aa, 4.59, 61/28 | Molybdenum cofactor biosynthesis protein MoaE | Htur_3481, 275 aa, 62% |
| Nmag_2218, 190 aa, 4.36, 34/15 | Molybdenum cofactor biosynthesis protein MoaB | Htur_0423, 195 aa, 85% |
| Nmag_2338, 228 aa, 4.33, 42/19 | Molybdopterin binding domain protein | Htur_1389, 228 aa, 75% |
| Nmag_2721, 217 aa, 4.06, 44/12 | Molybdenum cofactor biosynthesis protein MoaB | Htur_0764, 246 aa, 58% |
| **NADH-quinone/plastoquinone oxidoreductase complexes** | | |
| Nmag_3245, 506 aa, 4.52, 34/17 | NADH dehydrogenase-like complex subunit N | Htur_1107, 511 aa, 78% |
| Nmag_3246, 509 aa, 4.43, 37/16 | NADH dehydrogenase-like complex subunit M | Htur_1108, 509 aa, 85% |
| Nmag_3247, 678 aa, 4.88, 53/31 | NADH dehydrogenase-like complex subunit L | Htur_1109, 681 aa, 81% |
| Nmag_3248, 101 aa, 6.71, 5/5 | NADH dehydrogenase-like complex subunit K | Htur_1110, 101 aa, 92% |
| Nmag_3249, 162 aa, 3.83, 26/7 | NADH dehydrogenase-like complex subunit J2 | Htur_1111, 156 aa, 58% |
| Nmag_3250, 96 aa, 4.14, 10/1 | NADH dehydrogenase-like complex subunit J1 | Htur_1112, 91 aa, 75% |
| Nmag_3251, 153 aa, 4.85, 25/16 | NADH dehydrogenase-like complex subunit I | Htur_1113, 153 aa, 93% |
| Nmag_3252, 371 aa, 4.75, 26/15 | NADH dehydrogenase-like complex subunit H | Htur_1114, 364 aa, 82% |
| Nmag_3253, 560 aa, 4.31, 117/47 | NADH dehydrogenase-like complex subunit CD | Htur_1115, 552 aa, 82% |
| Nmag_3254, 235 aa, 4.90, 37/26 | NADH dehydrogenase-like complex subunit B | Htur_1116, 235 aa, 92% |
| Nmag_3255, 149 aa, 5.09, 14/11 | NADH dehydrogenase-like complex subunit A | Htur_1117, 137 aa, 89% |
| **Gluconeogenesis** | | |
| Nmag_0472, 898 aa, 4.54, 177/100 | Phosphoenolpyruvate carboxylase | Htur_4721, 896 aa, 84% |
| Nmag_2313, 290 aa, 4.21, 53/22 | Fructose-1,6-bisphosphatase | Htur_1165, 289 aa, 75% |
| Nmag_2598, 785 aa, 4.24, 156/65 | Phosphoenolpyruvate synthase | Htur_0801, 780 aa, 89% |
| Nmag_3507, 503 aa, 4.22, 110/43 | Phosphoenolpyruvate carboxykinase | Htur_1510, 503 aa, 81% |
| Nmag_3827, 288 aa, 4.16, 47/15 | Fructose-1,6-bisphosphatase | Htur_1165, 289 aa, 61% |
| Nmag_3829, 760 aa, 4.16, 157/61 | Phosphoenolpyruvate synthase | None (PERMA_0568, 812 aa, 50%) |
| **Glycolysis** | | |
| Nmag_0429, 403 aa, 3.99, 82/23 | Enolase | Htur_2545, 401 aa, 89% |
| Nmag_0603, 214 aa, 3.98, 35/8 | Triosephosphate isomerase | Htur_1904, 214 aa, 91% |
| Nmag_1259, 237 aa, 4.06, 44/14 | Triosephosphate isomerase | None (NP3716A, 230 aa, 48%) |
| Nmag_2141, 337 aa, 4.26, 67/26 | Glyceraldehyde-3-phosphate dehydrogenase, type II | Htur_0284, 337 aa, 89% |
| Nmag_2266, 507 aa, 4.18, 104/35 | Phosphoglycerate mutase, 2,3-bisphosphoglycerate-independent | Htur_0622, 507 aa, 88% |
| Nmag_2312, 264 aa, 4.30, 49/19 | Fructose-bisphosphate aldolase | Htur_1166, 264 aa, 89% |
| Nmag_2382, 311 aa, 4.81, 52/31 | Pyruvate-ferredoxin oxidoreductase β subunit | Htur_1443, 311 aa, 94% |
| Nmag_2383, 634 aa, 4.30, 128/48 | Pyruvate-ferredoxin oxidoreductase α subunit | Htur_1442 , 633 aa, 89% |
| Nmag_2605, 588 aa, 4.40, 109/56 | Pyruvate kinase | Htur_0844, 585 aa, 83% |
| Nmag_2711, 435 aa, 4.12, 81/27 | Phosphoglucose isomerase | Htur_0751, 433 aa, 83% |
| Nmag_3327, 404 aa, 4.25, 75/29 | Phosphoglycerate kinase | Htur_1600, 408 aa, 88% |
| Nmag_3794, 356 aa, 4.30, 60/26 | Glyceraldehyde-3-phosphate dehydrogenase, type I | None (HacjB3_14550, 349 aa, 68%) |
| Nmag_3795, 402 aa, 4.20, 84/28 | Phosphoglycerate kinase | Htur_1600, 408 aa, 42% |
| **Ribose-related enzymes** | | |
| Nmag_1339, 299 aa, 4.34, 55/23 | 6-phosphogluconate dehydrogenase, decarboxylating | Htur_3388, 299 aa, 92% |
| Nmag_1743, 291 aa, 4.26, 48/19 | Ribose-phosphate pyrophosphokinase | Htur_0087, 284 aa, 79% |
| Nmag_1945, 250 aa, 4.26, 42/17 | Ribose 5-phosphate isomerase | Htur_3758, 235 aa, 76% |
| Nmag_2967, 233 aa, 4.22, 45/15 | Deoxyribose-phosphate aldolase | Htur_0572, 211 aa, 76% |
| **Tricarboxylic acid cycle** | | |
| Nmag_0549, 290 aa, 4.43, 49/20 | Oxoglutarate--ferredoxin oxidoreductase beta subunit | Htur_1985, 287 aa, 96% |
| Nmag_0550, 583 aa, 4.29, 116/51 | Oxoglutarate--ferredoxin oxidoreductase alpha subunit | Htur_1986, 585 aa, 91% |
| Nmag_0736, 290 aa, 4.08, 51/15 | Succinate-CoA ligase α subunit | Htur_2061, 290 aa, 93% |
| Nmag_0737, 385 aa, 4.00, 82/25 | Succinate-CoA ligase β subunit | Htur_2062, 384 aa, 90% |
| Nmag_1336, 656 aa, 4.08, 135/41 | Aconitate hydratase | Htur_3383, 657 aa, 94% |
| Nmag_1476, 382 aa, 4.32, 73/33 | Citrate synthase | Htur_3036, 382 aa, 87% |
| Nmag_1974, 304 aa, 4.28, 56/24 | Malate dehydrogenase | Htur_3728, 304 aa, 91% |
| Nmag_2420, 481 aa, 4.65, 71/40 | Citrate synthase | Htur_3036, 382 aa, 39% |
| Nmag_2423, 377 aa, 4.75, 59/35 | Citrate synthase | Htur_3036, 382 aa, 41% |
| Nmag_2939, 434 aa, 4.32, 93/42 | Isocitrate dehydrogenase, NADP-dependent | Htur_0547, 425 aa, 80% |
| Nmag_2989, 920 aa, 4.16, 189/59 | Aconitate hydratase | Htur_1679, 922 aa, 92% |
| Nmag_3125, 143 aa, 5.43, 10/7 | Succinate dehydrogenase subunit C (cytochrome b-556) | Htur_0933, 131 aa, 81% |
| Nmag_3126, 121 aa, 9.40, 3/5 | Succinate dehydrogenase subunit D (membrane anchor) | Htur_0934, 121 aa, 83% |
| Nmag_3127, 292 aa, 4.75, 60/34 | Succinate dehydrogenase subunit B (iron-sulfur protein) | Htur_0935, 293 aa, 87% |
| Nmag_3128, 610 aa, 4.47, 115/49 | Succinate dehydrogenase subunit A (flavoprotein) | Htur_0936, 612 aa, 90% |
| Nmag_3595, 480 aa, 4.46, 87/39 | Fumarate hydratase | Htur_1812, 469 aa, 93% |
| **Other metabolic functions** | | |
| Nmag_0008, 322 aa, 4.41, 65/19 | Xylose isomerase domain protein TIM barrel | Htur_1849, 322 aa, 86% |
| Nmag_0029, 337 aa, 4.70, 57/31 | D-isomer specific 2-hydroxyacid dehydrogenase NAD-binding protein | Htur_1883, 325 aa, 81% |
| Nmag_0194, 122 aa, 4.50, 25/11 | Methylglyoxal synthase | Htur_2192, 122 aa, 80% |
| Nmag_0394, 607 aa, 4.54, 97/44 | Inositol monophosphatase | Htur_3821, 593 aa, 72% |
| Nmag_0457, 1014 aa, 4.48, 172/89 | FAD-dependent oxidoreductase | Htur_4692, 996 aa, 66% |
| Nmag_0687, 292 aa, 4.30, 55/26 | Inositol monophosphatase | Htur_2074, 270 aa, 71% |
| Nmag_0732, 418 aa, 4.63, 72/33 | Ribulose bisphosphate carboxylase, type III | None (HVO_0970, 414 aa, 70%) |
| Nmag_0777, 415 aa, 4.36, 73/28 | Myo-inositol-1-phosphate synthase | None (HVO_B0213, 398 aa, 63%) |
| Nmag_0779, 276 aa, 3.97, 70/13 | Xylose isomerase domain protein TIM barrel | None (HVO_B0210, 291 aa, 62%) |
| Nmag_0781, 282 aa, 4.29, 59/23 | Xylose isomerase domain protein TIM barrel | None (HVO_B0208, 283 aa, 67%) |
| Nmag_0807, 520 aa, 4.29, 108/39 | Aldehyde dehydrogenase | Htur_1200, 527 aa, 84% |
| Nmag_0809, 351 aa, 4.08, 63/22 | Arginase | Htur_2266, 306 aa, 58% |
| Nmag_0818, 208 aa, 4.26, 37/12 | D-isomer specific 2-hydroxyacid dehydrogenase NAD-binding protein | None (Mfer_0845, 526 aa, 42%) |
| Nmag_0821, 405 aa, 4.31, 81/33 | D-gluconate dehydratase | Htur_2654, 412 aa, 53% |
| Nmag_0822, 370 aa, 4.49, 68/33 | Mandelate racemase/muconate lactonizing protein | None (Hlac_2580, 369 aa, 76%) |
| Nmag_0931, 478 aa, 4.32, 92/38 | Glycerol-3-phosphate dehydrogenase, subunit C | Htur_2960, 448 aa, 84% |
| Nmag_0932, 449 aa, 4.34, 74/30 | Glycerol-3-phosphate dehydrogenase, subunit B | Htur_2961, 442 aa, 77% |
| Nmag_0933, 556 aa, 4.50, 104/52 | Glycerol-3-phosphate dehydrogenase, subunit A | Htur_2962, 556 aa, 90% |
| Nmag_0934, 527 aa, 4.18, 95/36 | Glycerol kinase | Htur_2963, 513 aa, 88% |
| Nmag_1145, 412 aa, 4.52, 81/37 | D-gluconate dehydratase | Htur_2654, 412 aa, 93% |
| Nmag_1154, 752 aa, 4.16, 170/55 | Alpha amylase catalytic region | Htur_2652, 682 aa, 58% |
| Nmag_1155, 412 aa, 4.42, 82/34 | D-gluconate dehydratase | Htur_2654, 412 aa, 87% |
| Nmag_1323, 376 aa, 4.59, 67/28 | Sarcosine oxidase | Htur_0024, 381 aa, 66% |
| Nmag_1334, 306 aa, 4.47, 56/25 | Luciferase-type oxidoreductase | Htur_3382, 307 aa, 79% |
| Nmag_1352, 548 aa, 4.32, 117/45 | Sulfatase | Htur_3376, 509 aa, 73% |
| Nmag_1354, 621 aa, 4.19, 137/48 | Sulfatase | Htur_3375, 474 aa, 66% |
| Nmag_1363, 159 aa, 4.09, 25/8 | Pyruvoyl-dependent arginine decarboxylase | Htur_3361, 158 aa, 80% |
| Nmag_1506, 517 aa, 4.48, 110/42 | UPF0447 protein | Htur_3475, 518 aa, 80% |
| Nmag_1529, 499 aa, 4.45, 98/40 | Sulfatase | Htur_3492, 498 aa, 75% |
| Nmag_1575, 327 aa, 4.11, 68/21 | D-isomer specific 2-hydroxyacid dehydrogenase NAD-binding protein | Htur_3563, 309 aa, 72% |
| Nmag_1763, 501 aa, 4.15, 97/31 | Argininosuccinate lyase | Htur_0330, 554 aa, 76% |
| Nmag_1764, 409 aa, 4.15, 87/29 | Argininosuccinate synthase | Htur_0331, 429 aa, 88% |
| Nmag_1773, 270 aa, 4.21, 43/14 | Phospholipase/Carboxylesterase | Htur_0274, 222 aa, 70% |
| Nmag_1999, 119 aa, 4.20, 22/18 | NifU C-terminal domain protein | Htur_3685, 123 aa, 91% |
| Nmag_2155, 503 aa, 4.40, 79/32 | Aldehyde dehydrogenase | Htur_0356, 503 aa, 89% |
| Nmag_2198, 193 aa, 4.20, 41/9 | Isochorismatase family protein | Htur_0399, 192 aa, 80% |
| Nmag_2244, 245 aa, 4.42, 46/18 | Creatininase | Htur_0439, 246 aa, 77% |
| Nmag_2289, 346 aa, 4.51, 65/33 | Mandelate racemase/muconate lactonizing protein | Htur_0594, 345 aa, 75% |
| Nmag_2321, 466 aa, 4.23, 96/39 | Aldehyde dehydrogenase | Htur_1159, 466 aa, 83% |
| Nmag_2331, 581 aa, 4.26, 120/42 | Aldehyde dehydrogenase | Htur_1200, 527 aa, 85% |
| Nmag_2387, 383 aa, 4.50, 66/31 | Chorismate synthase | Htur_1434, 383 aa, 97% |
| Nmag_2427, 620 aa, 4.10, 109/36 | Phospholipase-like protein | Htur_1506, 622 aa, 61% |
| Nmag_2465, 485 aa, 4.16, 84/31 | Aldehyde dehydrogenase | Htur_4196, 483 aa, 51% |
| Nmag_2513, 131 aa, 4.12, 29/10 | NifU C-terminal domain protein | Htur_3067, 129 aa, 84% |
| Nmag_2574, 455 aa, 4.83, 74/50 | Amine oxidase | Htur_1312, 446 aa, 62% |
| Nmag_2624, 254 aa, 4.29, 48/15 | Creatininase | Htur_0910, 263 aa, 44% |
| Nmag_2632, 379 aa, 5.03, 57/31 | Sarcosine oxidase | Htur_0024, 381 aa, 52% |
| Nmag_2691, 342 aa, 4.46, 60/25 | Luciferase family oxidoreductase, group 1 | Htur_3497, 346 aa, 64% |
| Nmag_2893, 456 aa, 4.41, 70/34 | Amine oxidase | None (ROP_04530, 450 aa, 42%) |
| Nmag_2918, 497 aa, 4.22, 80/25 | Aldehyde dehydrogenase | Htur_0525, 490 aa, 78% |
| Nmag_2923, 327 aa, 4.44, 56/29 | FAD-dependent oxidoreductase | Htur_0532, 326 aa, 82% |
| Nmag_3076, 408 aa, 4.37, 74/34 | Glycerol-3-phosphate dehydrogenase subunit A | Htur_5204, 424 aa, 72% |
| Nmag_3103, 273 aa, 4.55, 48/15 | Creatininase | Htur_0910, 263 aa, 85% |
| Nmag_3208, 195 aa, 4.46, 37/17 | Isochorismatase family protein | Htur_3075, 212 aa, 68% |
| Nmag_3397, 108 aa, 4.48, 27/16 | Chorismate mutase | Htur_5134, 108 aa, 85% |
| Nmag_3488, 489 aa, 4.49, 86/42 | Aldehyde dehydrogenase | Htur_1629, 496 aa, 85% |
| Nmag_3570, 554 aa, 4.45, 107/51 | Isochorismate synthase | Htur_1784, 451 aa, 59% |
| Nmag_3637, 285 aa, 4.31, 53/15 | Pirin domain protein | Htur_2629, 250 aa, 66% |
| Nmag_3658, 360 aa, 4.56, 63/34 | Mandelate racemase/muconate lactonizing protein | None (HVO_A0429A, 351 aa, 51%) |
| Nmag_3760, 392 aa, 4.33, 78/32 | Creatinase | Htur_2943, 392 aa, 87% |
| Nmag_3764, 1070 aa, 4.37, 194/79 | FAD-dependent oxidoreductase | Htur_2940, 1055 aa, 80% |
| Nmag_3765, 323 aa, 4.64, 52/21 | D-isomer specific 2-hydroxyacid dehydrogenase NAD-binding protein | Htur_2939, 325 aa, 84% |
| Nmag_3825, 451 aa, 4.40, 88/35 | Sulfatase | Htur_3375, 474 aa, 54% |
| Nmag_3838, 503 aa, 4.00, 93/23 | Aldehyde dehydrogenase | Htur_4061, 505 aa, 56% |
| Nmag_3841, 503 aa, 4.21, 88/28 | Aldehyde dehydrogenase | Htur_4591, 486 aa, 40% |
| Nmag_3900, 335 aa, 4.44, 67/33 | Luciferase family protein | Htur_2881, 334 aa, 80% |
| Nmag_3925, 388 aa, 4.51, 71/34 | Mandelate racemase/muconate lactonizing protein | Htur_2826, 391 aa, 70% |
| Nmag_3926, 409 aa, 4.59, 72/39 | Mandelate racemase/muconate lactonizing protein | Htur_3874, 401 aa, 39% |
| Nmag_3936, 410 aa, 4.40, 75/30 | D-gluconate dehydratase | Htur_4388, 423 aa, 76% |
| Nmag_3988, 320 aa, 4.65, 50/25 | D-isomer specific 2-hydroxyacid dehydrogenase NAD-binding protein | Htur_2328, 312 aa, 47% |
| Nmag_4042, 325 aa, 4.27, 56/21 | Urea amidolyase related protein | None (HacjB3_02220, 323 aa, 66%) |
| Nmag_4043, 300 aa, 4.07, 65/18 | Allophanate hydrolase subunit 1 | None (HacjB3_02225, 300 aa, 66%) |
| Nmag_4055, 511 aa, 4.35, 103/40 | Aldehyde dehydrogenase | Htur_1200, 527 aa, 84% |
| Nmag_4095, 531 aa, 4.52, 85/49 | Aldehyde dehydrogenase | None (Hlac_1582, 551 aa, 61%) |
| *When a homolog is not present in *Haloterrigena turkmenica* DSM 5511, the closest homolog from other archaea or bacteria is given in parenthesis. | | |
